# Efficient Consecutive Synthesis of Fluorinated Isoflavone Analogs, X-Ray Structures, Hirshfeld Analysis, and Anticancer Activity Assessment

**DOI:** 10.3390/molecules30040795

**Published:** 2025-02-09

**Authors:** Mohammed Salah Ayoup, Malak Daqa, Yousef Salama, Rand Hazzam, Mohammed B. Hawsawi, Saied M. Soliman, Nawaf Al-Maharik

**Affiliations:** 1 Department of Chemistry, College of Science, King Faisal University, Al-Ahsa 31982, Saudi Arabia; 2 Department of Chemistry, Science College, An-Najah National University, P.O. Box 7, Nablus 00970, Palestine; malak.d@najah.edu (M.D.); rand.hazzam@najah.edu (R.H.); 3 An-Najah Center for Cancer and Stem Cell Research, Faculty of Medicine and Health Sciences, An-Najah National University, P.O. Box 7, Nablus 00970, Palestine; yousef.ut@najah.edu; 4 Department of Chemistry, Faculty of Science, Umm Al-Qura University, Makkah 21955, Saudi Arabia; mbhawsawi@uqu.edu.sa; 5 Department of Chemistry, Faculty of Science, Alexandria University, Alexandria 21321, Egypt; saeed.soliman@alexu.edu.eg

**Keywords:** 4′-fluoroisflavones, 7-*O*-carboxmethyl-4′-fluoroisoflavone, XRD crystallography, anticancer, MCF7 cancer cells

## Abstract

The synthesis of 7-*O*-carboxymethyl-4′-fluoroisoflavone **4** and 7-*O*-carboxymethyl-4′-fluoro-2-trifluormethylisoflavone **7** involved the cyclization of 2,4-dihydroxy-4′-fluorodeoxybenzoin **1**, followed by 7-*O*-alkylation with methyl bromoacetate and subsequent acid-catalyzed hydrolysis. The structures of the novel compounds were validated using a range of techniques, including XRD crystallography (^1^H, ^19^F, ^13^C)-NMR, and IR. Only interhalogen contacts were detected in **5**, while they were completely lacking in **2** and **4**, owing to the presence of crystalline ethanol in the crystal structure. The %F…F in **5** was 12.2% based on Hirshfeld calculations. The aromatic π-π stacking interactions were important only in **2** and **4** but not observed in **5**. Isoflavones **4**, **5**, and **7** displayed anticancer activity against MCF-7 cancer cells, with IC_50_ values of 13.66, 15.43, and 11.73 µM, respectively.

## 1. Introduction

**Figure 1:**
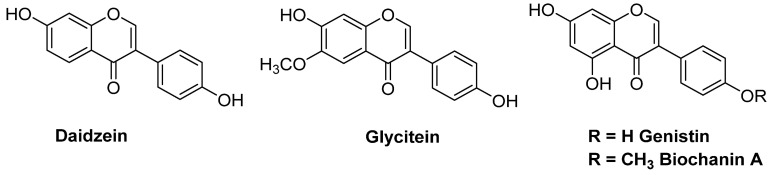
The predominant isoflavones found in soy.

Scientists and health enthusiasts have expressed significant interest in isoflavones owing to their fascinating properties and prospective health benefits. Isoflavones are the primary subgroup of a broader category of isoflavonoids, which collectively form a subset of flavonoids. Unlike other flavonoid subclasses, isoflavones demonstrate a restricted distribution throughout 20 plant families, attributable to the scarce presence of isoflavone synthase, which is a crucial enzyme involved in the synthesis of isoflavones [1,2,3]. Soybeans serve as the primary source of isoflavones, predominantly in glycosylated forms, including the following prominent isoflavone aglycones: genistein, daidzein, glycitein, and biochanin A (Figure 1).

Plant isoflavones can be classified into three categories: isoflavone glycosides, prenylated derivatives, and isoflavones of basic substitution patterns such as hydroxy, methoxy, methylenedioxy, hydroxymethyl, and acetyl patterns [3]. Furthermore, in whole-cell biotransformation, microbes such as fungi and actinomycetes have been used to synthesize non-plant-chlorinated isoflavones, as well as simple and complex isoflavones [3,4,5]. Isoflavones are well known for their high pharmacological activity and low toxicity, making them a focus of interest in medicinal research and development [5,6]. They play an important role in plant growth and development, as well as in the prevention of bacterial infections and illnesses [7,8,9]. Notably, they demonstrate a diverse range of biological capacities, encompassing antibacterial [10], antifungal [11], antiviral [12], anticancer [13,14,15], anti-inflammatory [16], antiplatelet [17], and antiaging [18] properties. Isoflavones have estrogenic effects as they share structural and functional similarities with 17β-estradiol, the primary form of estrogen in the human body. They predominantly bind to estrogen receptors (ERs), specifically ER-α and ER-β [19]. Epidemiological studies have revealed that Asian women have a reduced incidence of breast cancer, the most common malignant tumor among women, in contrast to their Western counterparts, which is a difference ascribed to their elevated consumption of phytoestrogens [20]. Thus, phytoestrogens may function as a viable strategy in the prevention and treatment of breast cancer via mechanisms such as estrogen receptor modulation and anti-angiogenesis [21].

Despite the potential of natural isoflavones for pharmaceutical development, their advancement in the drug discovery process is impeded by various issues, including inadequate solubility, bioavailability, and selectivity [22]. Various efforts have been made to improve the therapeutic effectiveness of isoflavones by including functional groups such as halogens, amino groups, and amino acids [22]. Fluorinated pharmaceuticals have considerable emphasis in pharmaceutical science, with around 20% of medications containing one or more fluorine atoms [23]. The diminutive atomic size and elevated electronegativity of fluorine significantly influence the characteristics of medicinal compounds. These characteristics encompass drug–target interactions, selectivity, metabolic stability, acidity or basicity, membrane permeability, and toxicity. The fluorine atom can function as a bioisostere for amino and hydroxyl groups, as well as hydrogen atoms, modifying the processes of pharmacological action and their impacts on biological systems [24]. The incorporation of fluorine into an aryl ring or other π-system enhances lipophilicity due to the resonance donation of fluorine’s lone-pair electrons [25].

To further advance our endeavors in identifying potential therapeutic candidates, we introduced the synthesis of the novel fluorinated isoflavone-bearing carboxymethyl group at the 7-*O*-position. The anticancer potential of these compounds was subsequently assessed against the MCF-7, B16F10, and MEF-1 cell lines. An analysis of this unique chemical’s structure was conducted utilizing IR, (^1^H, ^19^F, ^13^C)-NMR, HR ESI-MS, and single-crystal XRD.

## 2. Results

**Scheme 1:**
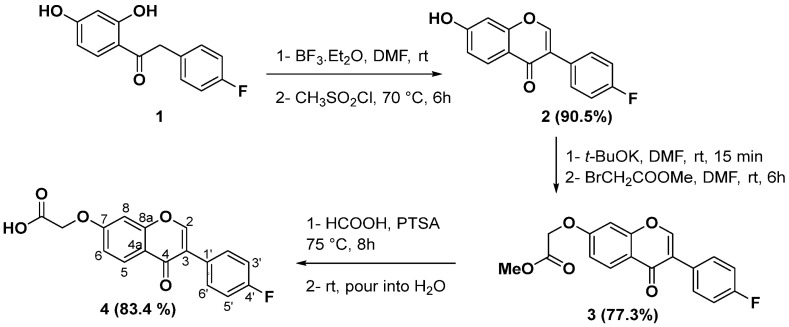
Total synthetic pathway of 2-((3-(4-fluorophenyl)-4-oxo-4*H*-chromen-7-yl)oxy)acetic acid **4**.

**Scheme 2:**
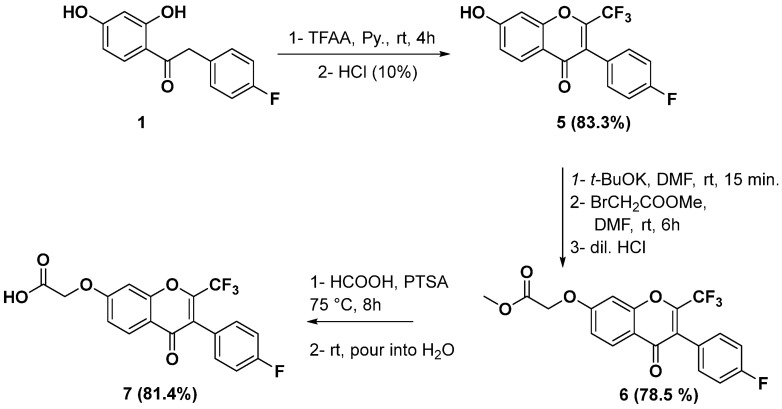
Total synthetic pathway of 2-((3-(4-fluorophenyl)-4-oxo-2-(trifluoromethyl)-4H-chromen-7-yl)oxy)acetic acid **7**.

Scheme 1 and Scheme 2 illustrate the synthetic procedures for the new fluorinated isoflavone **4** and **7** analogs, starting with 2,4-dihydroxy-4′-fluorodeoxybenzoin **1**. A highly efficient and pure synthesis of 4′-fluoro-7-hydroxyisoflavone **2** was achieved by formylating and cyclizing deoxybenzoin **1** using dry DMF in the presence of BF_3_.Et_2_O and methanesulfonyl chloride (CH_3_SO_2_Cl) at a temperature of 70 °C [26]. Deprotonation of the 7-OH group of isoflavone **2** with non-nucleophilic potassium *tert*-butoxide (*t*-BuOK) at ambient temperature under an N_2_ atmosphere generated the phenoxide. Subsequently, the phenoxide was treated with methyl bromoacetate to produce methyl 2-((3-(4-fluorophenyl)-4-oxo-4*H*-chromen-7-yl)oxy)acetate **3** in excellent yields. The desired acid **4** was obtained in high yield and purity by subjecting ester **3** to *p*-toluenesulfonic acid-catalyzed hydrolysis in formic acid at 75 °C.

Hydroxyl groups were acylated in deoxybenzoin **1** with an excess of trifluoroacetic anhydride (TFAA) in dry pyridine under cooling, followed by the Baker–Venkataraman rearrangement and Allan–Robinson reaction, which afforded 3-(4-fluorophenyl)-7-hydroxy-2-(trifluoromethyl)-4*H*-chromen-4-one **5** in high yield (Scheme 2). After being treated with *t*-BuOK, the latter chemical **5** was subjected to 7-*O*-alkylation with methyl bromoacetate, resulting in the formation of methyl 2-((3-(4-fluorophenyl)-4-oxo-2-(trifluoromethyl)-4*H*-chromen-7-yl)oxy)acetate **6**. Upon treatment with a catalytic quantity of *p*-toluenesulfonic acid in formic acid at 75 °C, this compound produced the intended acid with a high yield and purity. The structures of the synthesized compounds were confirmed using IR, ^1^H NMR, ^13^C NMR (DEPTQ), HRMS and ^19^F NMR spectroscopy. IR spectra exhibited a broad band in the range of 3262–3204 cm⁻¹, indicating the presence of phenolic OH groups for compounds **2** and **5**; additionally, the C=O group manifested as a sharp band in the region of 1763–1624 cm⁻¹ for compounds **2**–**7**.

The structures of the synthesized compounds were confirmed using IR, ^1^H NMR, ^13^C NMR (DEPTQ), and ^19^F NMR spectroscopy. IR spectra exhibited a broad band in the range of 3262–3204 cm⁻¹, indicating the presence of phenolic OH groups for compounds **2** and **5**; additionally, the C=O group manifested as a sharp band in the region of 1763–1624 cm⁻¹ for compounds **2–7**. The ^1^H NMR spectra of the final product **4** exhibited six resonances corresponding to eight aromatic protons and one peak for methylene protons at 4.91 ppm. A singlet at 8.49 ppm was assigned to H-2. A doublet of doublets for two protons (H-2′,6′) at 7.63 ppm, with ^3^*J*_H2′-H3′_ = 8.7 Hz and ^4^*J*_H2′-F_ = 5.6 Hz, and another doublet of doublets appearing as a triplet for two protons (H-3′,5′) at 7.14 ppm, with ^3^*J*_H2′-H3′_ = 8.7 Hz and ^3^*J*_H2′-F_ = 8.7 Hz, were designated. The DEPTQ spectra displayed fifteen peaks, and the positioning of the F atom at the 4-position was validated by the ^1^*J*_C4′,F_ value of 244 Hz, in conjunction with the ^2^*J*_C,F_, ^3^*J*_C,F_, and ^4^*J*_C,F_ values of 21.2, 8.2, and 3.1 Hz, respectively, which corresponded with the expected values. The ^1^H NMR of isoflavone **7** was distinguished by the absence of the H-2 proton. The DEPTQ spectra for compound 7 indicated the positioning of the CF₃ group at the C-2 site, corroborated by a quartet between 119.3 and 147.0 ppm (C-2), with ^1^*J*_C,F_ = 275 Hz and ^2^*J*_C,F_ = 35.5 Hz. Finally, the FNMR of compounds 4 and 7 showed a singlet signal at *δ_F_* -114.22 (s, F) and two different signals at *δ_F_* −62.77 (C**F**_3_), −113.38 (1F) for compounds **4** and **7,** respectively.

## 3. Crystal Structure Description

**Figure 2:**
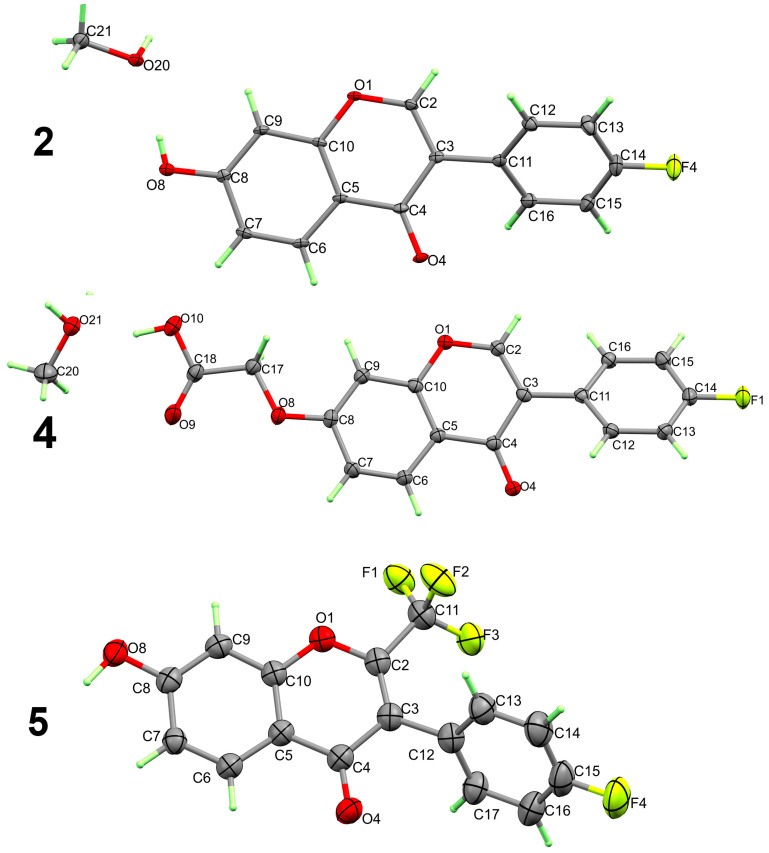
Structure with atom numbering for **2**, **4,** and **5**.

The X-ray structure of compounds **2**, **4**, and **5,** showing the atom numbering for the heavy atoms, is presented in Figure 2. The crystal parameters and some important bond distances and angles are depicted in Table 1 and Table 2, respectively. The structure of the three compounds comprised a planar 4*H*-chromen-4-one moiety with the *p-fluorophenyl* group as a substituent at C3. The 4*H*-chromen-4-one and fluorophenyl moieties were not in the same plane, whereas the mean planes between the two moieties showed different degrees of twists in the three compounds. The twist angles were 41.43, 32.30, and 62.22˚ for **2**, **4**, and **5**, respectively. The values of the twist angles were close to one another for both **2** and **4**, while it was significantly higher in **5**, which could be attributed to the presence of the trifluoromethyl substituent at C2. Hence, the presence of two bulky substituents in the neighborhood increased the twist angle between the 4H-chromen-4-one and fluorophenyl moieties due to the steric–electronic factors. Another interesting difference between the three compounds was the presence of a crystal solvent (methanol) in the case of compounds **2** and **4** but not in **5**, which had a significant impact on the molecular packing of the studied compounds.

**Figure 3:**
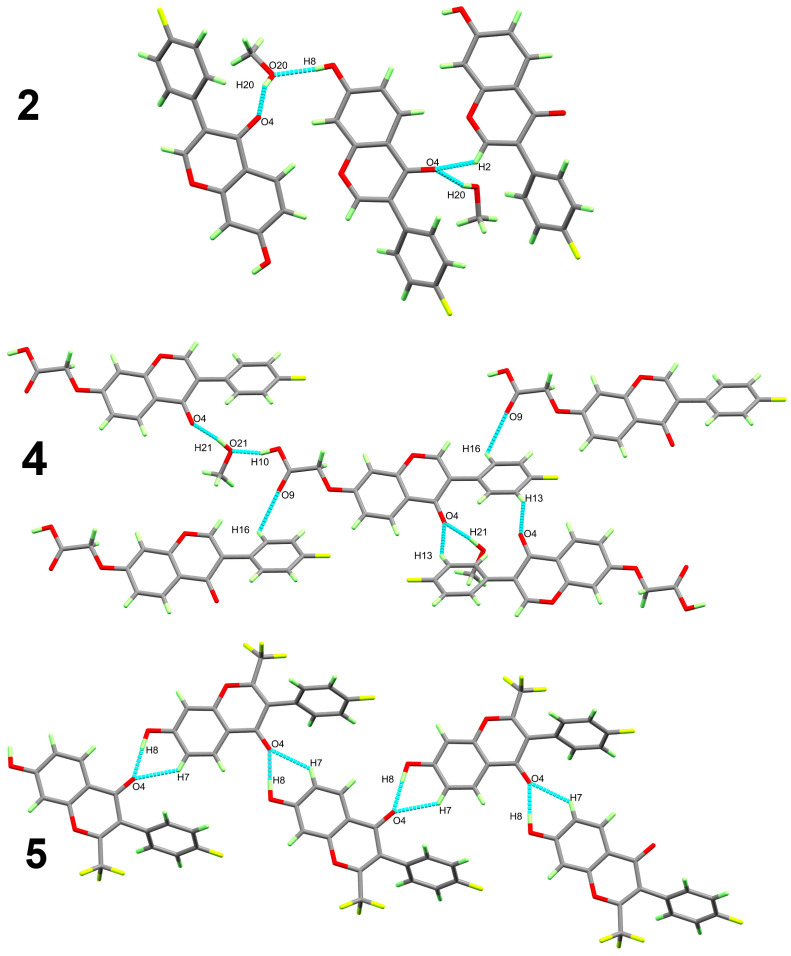
The hydrogen bond packing views for **2**, **4,** and **5**.

The molecular packing of the three compounds was controlled by the O-H…O hydrogen bonds shown in Figure 3. For **5**, the O-H, which is the hydrogen bond donor, makes with carbonyl oxygen (O4), which is the hydrogen acceptor for the significantly strong O8-H8…O4 hydrogen bond, where the hydrogen-to-acceptor (A) distance is only 1.70(2) Å, and the donor (D)-to-acceptor (A) distance is 2.664(3) Å. In addition, there is another relatively longer C7-H7…O4 interaction where the D…A distance is 3.092(3) Å. The presence of the methanol molecule as a crystal solvent in the case of **2** increases the number of O…H interactions. The O8-H8…O20 and O20-H20…O4 hydrogen bonds, which have H…A distances of 1.91(4) and 1.91(3) Å, respectively, in addition to the C2-H2…O4 interaction with the D…A distance of 3.121(4) Å are the important O…H contacts. Moreover, compound **4** not only has a methanol molecule as a crystal solvent but also has the polar COOH in its structure, which maximizes the hydrogen bonding interaction in this case (Table 3).

**Figure 4:**
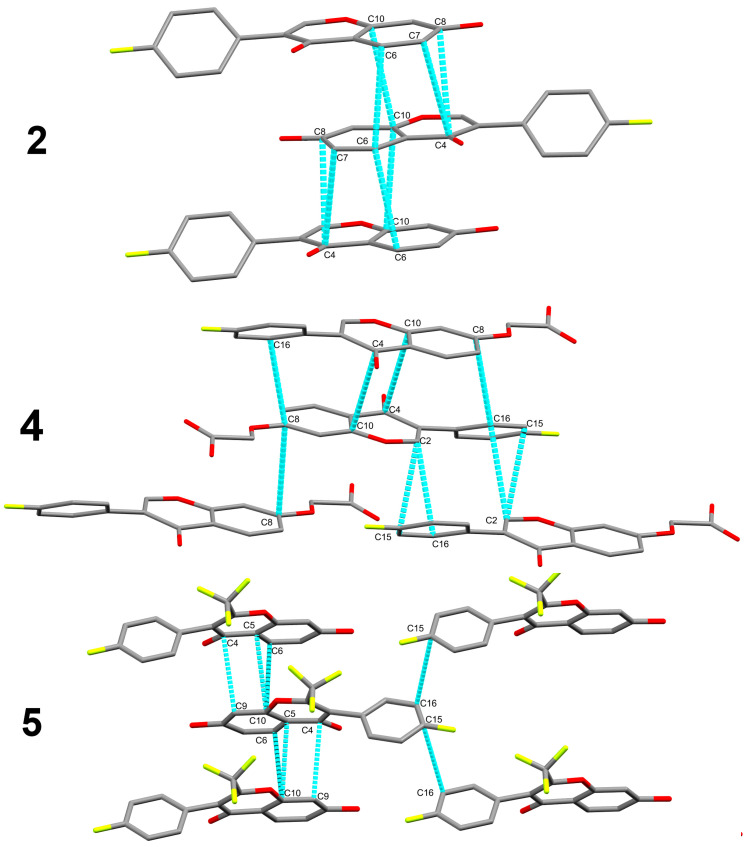
The π-π stacking interactions in **2**, **4,** and **5**.

In addition, the molecular packing of the studied compounds showed a number of π-π stacking interactions. The shortest π-π contacts in **2**, **4,** and **5** were the C7…C4 (3.408(5) Å), C8…C16 (3.280(2) Å), and C4…C9 (3.418(3) Å) interactions (Table 4). Many of these short aromatic–aromatic separations are shown in Figure 4, which support the supramolecular structure of the studied systems.

**Figure 5:**
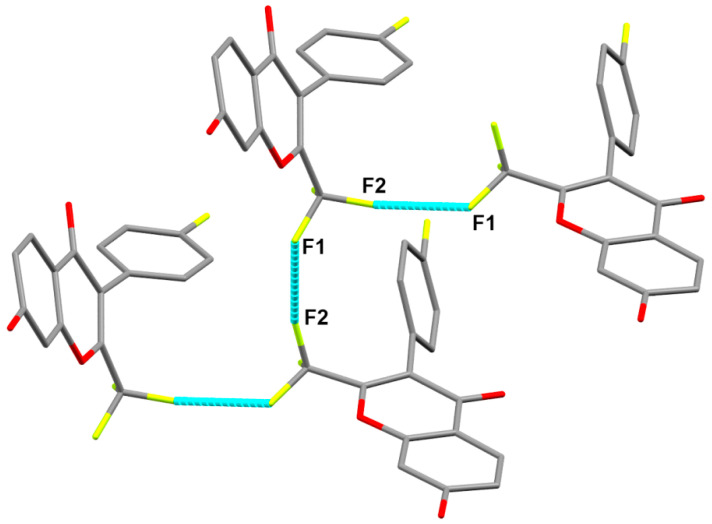
The interhalogen interaction in **5**.

It is worth noting that compound **5** is the only one that showed an interhalogen interaction (Figure 5). In this case, there was a short interhalogen F1…F2 interaction (2.837(2) Å) observed in **5**, which might be attributed to the absence of a solvent in the crystal structure. The presence of a methanol molecule as a crystal solvent could make further separation between the molecules in the crystals possible, which would decrease the possibility of interhalogen interactions, as found in **2** and **4**.

## 4. Topology Analysis

Topology analysis, with the aid of Hirshfeld calculations, provides a full picture of all possible intermolecular interactions in the crystal structure and plays an important role in crystal stability. Even contacts that contribute a small amount to the molecular packing could be analyzed using this tool [27,28]

**Figure 6:**
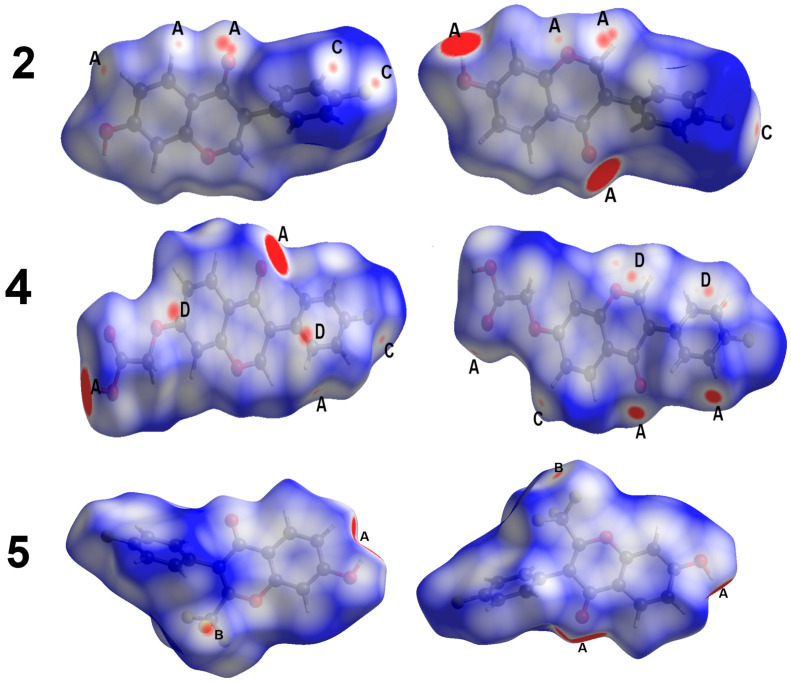
Hirshfeld d_norm_ maps showing important contacts in the studied compounds: O…H (A), F…F (B), F…H (C), and C…C (D).

The analysis of molecular packing was performed using Hirshfeld topology calculations. The red spots in the d_norm_ map are related to short significant contacts in the studied systems (Figure 6). In **5**, the O…H and the interhalogen F…F interactions were the most important, while in both **2** and **4**, the important contacts were the O…H and F…H interactions. Additionally, there are many important C…C interactions in **4** that are related to the π-π interactions, where the C2…C15 (3.328 Å) and C8…C16 (3.280 Å) interactions are the shortest (Table 5). In addition, O20…H8 (1.684 Å), O4…H21 (1.691 Å), and O4…H8 (1.683 Å) are the most important O…H contacts in **2, 4,** and **5**, respectively. F4…H15 (2.481 Å) in **2** is slightly shorter than F1…H7 (2.489 Å) in **4**.

**Figure 7:**
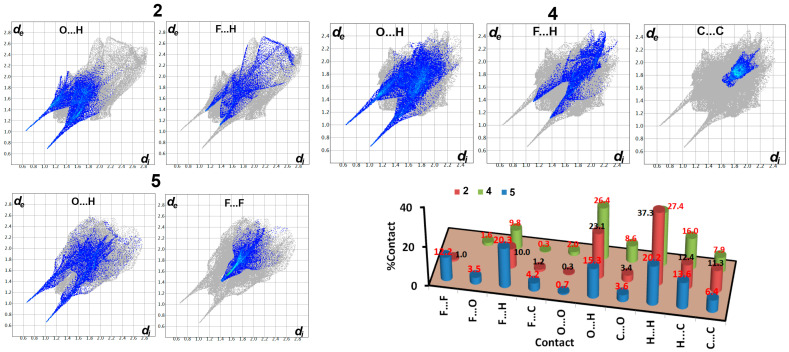
Fingerprint plots for the important contacts and %contacts in the studied compounds.

In addition, the fingerprint plots for the important contacts are presented in Figure 7. The fingerprint plot further indicated the importance of the contacts presented in Figure 6. Also, the area of the fingerprint plot gave the percentage of each contact. The %O…H contacts in compounds **2**, **4,** and **5** were 23.2, 26.4, and 15.3%, respectively, while %F…H contacts were 10.0 and 9.8% for **2** and **4**, respectively. For **5**, the %F…F contact was 12.2%, while for **4**, the %C…C contact was 7.9%.

**Figure 8:**
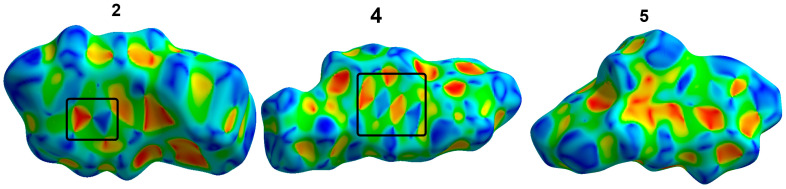
Shape index maps for the studied compounds.

In Figure 8, the shape index maps of the studied compounds are presented. No red/blue triangles are observed in the shape index map of **5,** while the opposite is true for **2** and **4**. Hence, the π-π stacking interactions were important only in compounds **2** and **4**. It is worth noting that the C…C contacts are relatively longer in **2** than those found in **4**. In the former, the shortest C…C contact distance is 3.408 Å.

## 5. Anticancer Activity

**Figure 9:**
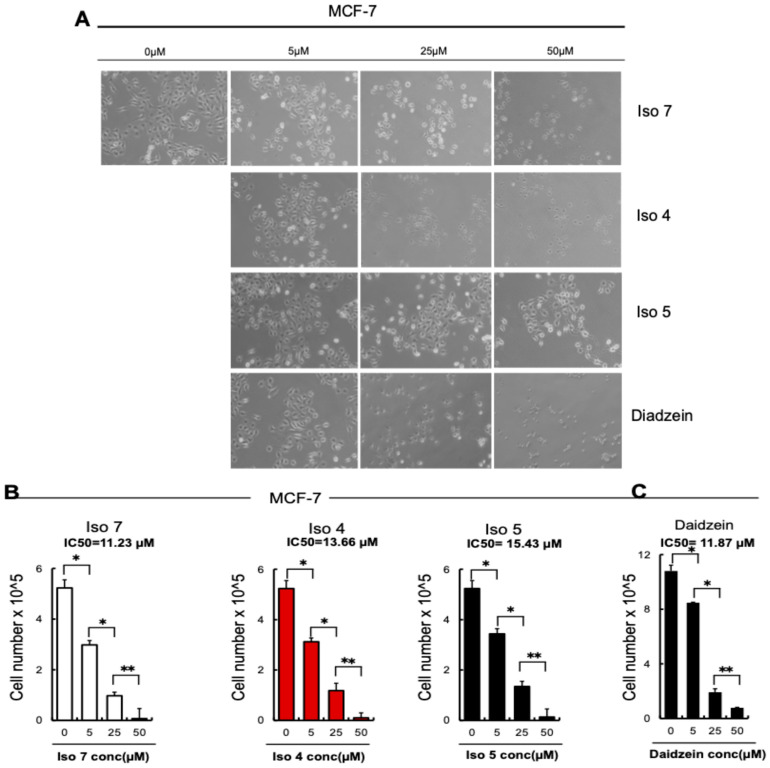
The synthetic isoflavones **4**, **5**, **7**, and daidzein were added in varying concentrations to MCF-7 cells. (**A**) Macroscopic images of MCF-7 cells under an inverted microscope after 24 h of treatment with **4**, **5**, **7**, and daidzein or DMSO as a control (n = 3 groups). (magnification 20×). (**B**,**C**). Isoflavones at the indicated concentrations were applied to human MCF-7 cells. After 24 h, viable cells (n = 3/group) were counted using Trypan blue. Data are expressed as mean ± SEM (unpaired Student’s *t*-test or one-way ANOVA * *p* < 0.05, ** *p* < 0.01). The linear regression curve was used to obtain the IC_50_ value, which is as follows: Y = a * X + b, IC_50_ = (0.5 − b)/a.

Breast cancer is the most prevalent cancer among women and the second most common disease globally [29,30]. The MCF-7 cell line is a typical model for human breast cancer that is dependent on estrogen [31]. MCF-7 cells, which are part of the luminal A molecular subtype, not only express progesterone receptors but also lack HER2/neu overexpression [32,33]. Upon exposure to the main isoflavones, genistein, and daidzein, which share structural and functional similarities with 17-estradiol, it was observed that the binding affinity of genistein to estrogen receptors of alpha (ERα) and beta (ERβ) was one to three orders of magnitude lower than that of 17-estradiol [34,35,36]. Studies were conducted to evaluate the impact of varying doses of isoflavones **7**, **4**, **5** and daidzein on cell proliferation in the highly metastatic murine B16F10 and human amelanotic MCF-7 breast cancer cell lines, as depicted in Figure 9A–C. The proliferation of MCF-7 cells was selectively suppressed by isoflavones, whereas B16F10 cells were not, in a concentration-dependent manner following 24 h of in vitro treatment. Conversely, the carrier treatment (DMSO) had no effect on cell proliferation (0 μM; Figure 9A–C).

**Figure 10:**
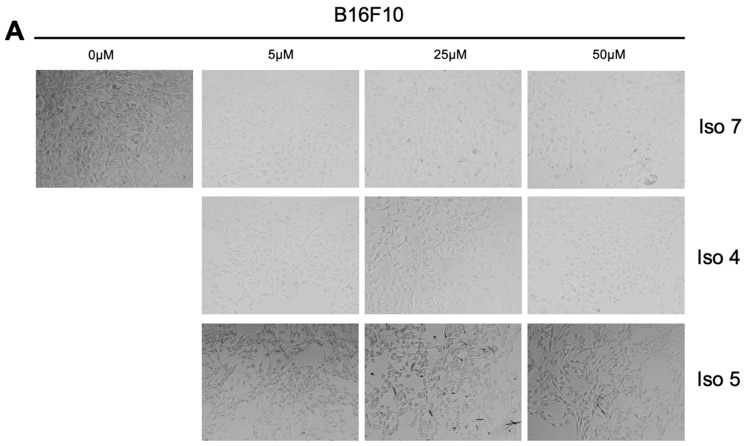

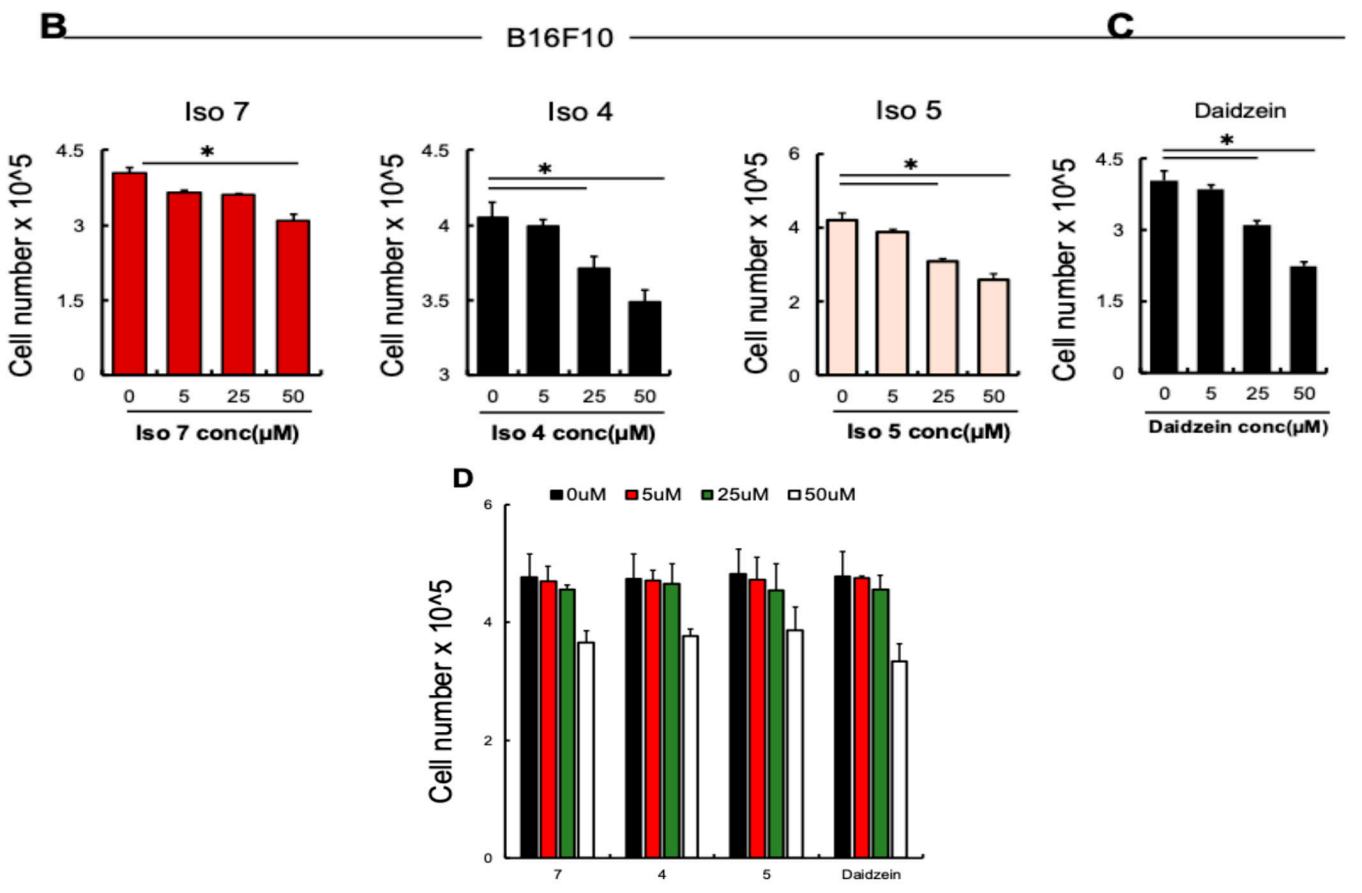
Various synthetic isoflavones, **4**, **5**, **7,** and daidzein, were added at varying concentrations to B16F10 cells. (**A**) Macroscopic images of B16F10 cells under an inverted microscope after treatment with different isoflavones (magnification 20×). (**B**,**C**). Isoflavones at the indicated concentrations were applied to human B16F10 cells. (**D**). The mouse embryonic fibroblast-1 (MEF-1) cell line was treated with the indicated concentrations of isoflavones **4**, **5**, **7,** and daidzein; after 24 h, viable cells (n = 3/group) were counted using Trypan blue. Data are expressed as mean ± SEM (unpaired Student’s *t*-test or one-way ANOVA * *p* < 0.05).

For MCF-7 cells, the doses of isoflavones **7**, **4, 5,** and daidzein that caused a 50% growth inhibition (IC_50_) were 11.23, 13.66, 15.43, and 11.87 μM, respectively, as shown in Figure 9B, C and Table 6. Daidzein and the three fluoroisoflavones studied showed significant activity against the MCF-7 cell line, activating the apoptotic pathway and inducing cell death in a dose-dependent manner. Our findings indicate that substituting 4′-OH with F and replacing the H-atom with the CF₃ group did not influence the anticancer efficacy of daidzein. Moreover, we also investigated the impact of the isoflavones presented to MCF-7 cells on B16F10 melanoma cells. Following 24 h of exposure to isoflavones **7**, **4**, **5**, and daidzein at the indicated concentration, as shown in Figure 10A–C, it was confirmed that isoflavones have little impact on B16F10 cells when administered at high doses. Unlike B16F10 melanoma cells, breast cancer (BC) and other hormone-sensitive malignancies rely on estrogen receptor (ER) signaling as a crucial control mechanism for cell division, population growth, and survival. Our results corroborate with previous research indicating that isoflavones, by virtue of their structural and functional resemblance to 17-estradiol, suppress the growth and cell migration of breast cancer cells [36]. Our findings support other studies which state that isoflavones inhibit the proliferation and migration of breast cancer cells [36]. It is interesting that our study showed isoflavones to have less of an effect on B16F10 melanoma cell lines. The dose-limiting toxicity of anticancer drugs is called cytotoxicity. According to earlier studies [37], mouse embryonic fibroblasts (MEF-1) can be used to evaluate the toxicity of non-malignant cells. When 25 μM of isoflavones was introduced, MEF-1 did not decrease proliferation (Figure 10D). These findings support the idea that isoflavones improve anti-proliferative effects on cancer cells, lowering cytotoxicity and requiring less medication.

IC_50_ values for the normal cell line MEF-1 and the cancer cell line MCF-7 are shown following 24 h of exposure to the specified isoflavones (Table 6). Half-maximal inhibitory concentration (IC_50_) values from three separate tests conducted in triplicate were used to display the data. Selectivity index: IC_50_ on normal cells/IC_50_ on cancer cells.

## 6. Experimental Section

### 6.1. Material and Measurement Details Are Reported in the Supplementary Information

All solvents and reagents were procured from [Sigma-Aldrich, St Louis, USA] and used without additional purification. ^1^H-, ^19^F-, and ^13^C-NMR (DEPTQ) were recorded in CDCl_3_ and DMSO-*d_6_* or CD_3_OD on a 400 and 500 MHz Bruker AV III spectrometer (Billerica, Massachusetts, USA). Chemical shifts are represented by the value *δ* (ppm), whereas coupling constants (*J*) are expressed in Hertz (Hz). The flash column chromatography employed Merck silica gel 60 with a particle size of 0.040–0.063 mm

### 6.2. Chemistry

#### 6.2.1. 4′-Fluoro7-hydroxyisoflavone (**2**)

BF_3_.Et_2_O (7.43 mL, 0.06 mol) was added to a solution of 1-(2,4-dihydroxyphenyl)-2-(4-fluorophenyl)ethan-1-one (**1**) (4.92 g, 0.02 mol) in DMF (30 mL) under N_2_. After 15 min, stirring at room temperature, a solution of methanesulfonyl chloride (4.67 mL, 0.06 mol) in DMF (10 mL) was added dropwise. After heating at 70 °C for 6 h, the reaction mixture was cooled to room temperature and poured into ice-cold aqueous sodium acetate (12 g/100 mL). The solid precipitate was filtered off and recrystallized from aqueous ethanol to produce the title compound **2** as a pale yellow solid (4.64 g, 0.0181 mol, 90.5%); Mp = 245–246 °C; FTIR (ν_max_/cm^−1^) 3262 br (OH), 1624, 1572, 1514, 1267, 1242; ^1^H NMR (500 MHz, DMSO-*d_6_*) *δ_H_* 10.87 (br s, 1H, 7-OH), 8.41 (s, 1H, H-2), 7.98 (d, *J* = 8.8 Hz, 1H, H-5), 7.62 (dd, *J* = 8.9, 5.4 Hz, 2H, H-2′,6′), 7.27 (dd, *J* = 8.9, 8.8 Hz, 2H, H-3′,5′), 6.96 (dd, *J* = 8.8, 2.2 Hz, 1H, H-6), 6.89 (d, *J* = 2.2 Hz, 1H, H-8). ^19^F NMR (471 MHz, DMSO-*d_6_*) *δ_C_* −114.37 (dt, *J* = 15.3, 5.7 Hz). ^13^C NMR (126 MHz, DMSO-*d_6_*) δ 174.8, 163.2, 162.6 (d, *J* = 245 Hz), 158.0, 154.4, 131.4 (d, *J* = 8.0 Hz), 128.9 (d, *J* = 3.7 Hz), 127.8, 123.0, 117.0, 115.8, 115.4 (d, *J* = 8.0 Hz), 102.7. HRMS calculated C_15_H_9_O_3_FNa (M + Na^+^) 279.0433, found 279.0421.

#### 6.2.2. Methyl 2-((3-(4-fluorophenyl)-4-oxo-4H-chromen-7-yl)oxy)acetate (**3**)

A solution of 4′-fluoro-7-hydroxyisoflavone (**2**) (0.4 g, 1.56 mmol) and potassium *tert*-butoxide (0.21 g, 1.87 mmol) in dry DMF (15 mL) was stirred at room temperature for 15 min under a nitrogen atmosphere. Thereafter, methyl bromoacetate (0.177 mL, 1.87 mmol) was added, and the mixture was stirred at room temperature for 6 h. The mixture was poured into ice water, neutralized with diluted HCl, and the yellow precipitate was filtered. The solid was subjected to flash chromatography to obtain the title compound **3** as a white solid (0.396 g, 0.00121 mol, 77.3%); Mp = 156–158 °C. FTIR (ν_max_/cm^−1^) ν_max_/cm^−1^ 1764, 1631, 1509, 1438, 1218, 1197. ^1^H NMR (400 MHz, CDCl_3_) *δ_H_* 8.24 (dd, *J* = 8.9, 0.9 Hz, 1H, H-5), 7.92 (s, 1H, H-2), 7.53 (dd, *J* = 8.9, 5.4 Hz, 2H, H-2′,6′), 7.12 (t, *J* = 8.8 Hz, 2H, H-3′,5′), 7.04 (dd, *J* = 8.9, 2.4 Hz, 1H, H-6), 6.84 (d, *J* = 2.4 Hz, 1H, H-8), 4.74 (s, 2H, -CH_2_-), 3.83 (s, 3H, CH_3_). ^19^F NMR (377 MHz, CDCl_3_) *δ_F_* −113.7 (s, 1F). ^13^C NMR (101 MHz, CDCl_3_) *δc* 175.4, 168.3, 162.7 (d, *J* = 245 Hz), 162.0, 157.7, 152.1, 130.7 (d, *J* = 8.3 Hz), 128.2, 127.7 (d, *J* = 8.3 Hz), 124.5, 119.1, 115.4 (d, *J* = 21.5 Hz), 114.5, 101.4, 65.3, 51.5. HRMS calculated C_18_H_13_O_5_FNa (M + Na^+^) 351.0645, found 351.0641.

#### 6.2.3. 2-((3-(4-Fluorophenyl)-4-oxo-4H-chromen-7-yl)oxy)acetic Acid **4**

The 4′-fluoro-7-O-(methoxycarbonylmethyl)isoflavone (**3**) (150 mg, 0.457 mmol) was dissolved in formic acid (5 mL), and a catalytic amount of *p*-toluenesulphonic acid (10 mg, 0.058 mmol) was added to the solution under a nitrogen atmosphere. The solution was stirred at 75 °C for 8 h. The completion of the reaction was monitored by TLC. After the disappearance of the starting material, the mixture was cooled to room temperature and diluted with distilled water. The white precipitate was collected, washed with water, and recrystallized from methanol to furnish **4** as a white solid (121 mg, 0.46 mmol, 83.4%), Mp = 244–245 °C. FTIR (ν_max_/cm^−1^) 2993 br (COOH), 1743, 1643, 1609, 1572, 1517, 1443, 1244, 1190. ^1^H NMR (400 MHz, DMSO-*d_6_*) *δ_H_* 8.49 (s, 1H, H-2), 8.05 (dd, *J* = 8.9, 2,4 Hz, 1H, H-5), 7.63 (dd, *J* = 8.7, 5.6 Hz, 2H, H-2′,6′), 7.28 (t, *J* = 8.7 Hz, 2H, H-3′,5′), 7.18 (d, *J* = 2.4 Hz, 1H, H-8), 7.14 (dd, *J* = 8.9, 2.4 Hz, 1H, H-6), 4.91 (s, 2H, -CH_2_-). ^19^F NMR (377 MHz, DMSO-*d_6_*) *δ_F_* −114.22 (s, F). ^13^C NMR (101 MHz, DMSO-*d_6_*) *δ_C_* 174.4, 169.5, 163.09, 162.3, 161.9 (d, *J* = 244 Hz), 157.3, 154.29, 131.0 (d, *J* = 8.2 Hz), 128.2 (d, *J* = 3.1 Hz), 127.1, 122.8, 117.92, 115.1, 115.0 2 (d, *J* = 7.5 Hz), 101.3, 65.0. HRMS calculated C_17_H_11_O_5_FNa (M + Na^+^) 337.0488, found 337.0485.

#### 6.2.4. 7-Hydroxy-3-(4-fluorophenyl)-2-(trifluoromethyl)-4H-chromen-4-one (**5**)

Trifluoroacetic anhydride (8.25 mL, 0.06 mol) was slowly added under a nitrogen atmosphere to a solution of 1-(2,4-dihydroxyphenyl)-2-(4-fluorophenyl)ethan-1-one (**1**) (4.92 g, 0.02 mol) in dry pyridine (15 mL). The mixture was stirred for 4 h, after which it was poured into a 10% HCl solution (100 mL). The mixture was stirred at 50 °C for 3 h. The precipitate was filtered and recrystallized from 70% ethanol to obtain the title compound **5** as a pale yellow solid (5.40 g, 0.0167 mol, 83.3%), Mp = 218–219 °C. FTIR (ν_max_/cm^−1^) 3204 br (OH), 1638, 1600, 1459, 1271, 1192, 1164, 1150; ^1^H NMR (400 MHz, CD_3_OD) *δ_H_* 8.01 (d, *J* = 8.8 Hz, 1H, H-5), 7.30 (dd, *J* = 8.8, 5.4 Hz, 2H, H-2′,6′), 7.19 (dd, *J* = 8.8, 8.8 Hz, 2H, H-3′,5′), 7.01 (dd, *J* = 8.8, 2.2 Hz, 1H, H-6), 6.92 (d, *J* = 2.2 Hz, 1H, H-8). ^19^F NMR (377 MHz, CD_3_OD) *δ_F_* −65.11 (3F), −115.24 (1F). ^13^C NMR (101 MHz, CD_3_OD) *δ_C_* 176.5, 164.5, 163.1 (d, *J* = 247 Hz), 157.2, 148.1 (d, *J* = 36 Hz), 131.8 (d, *J* = 8.6 Hz), 127.8, 125.5 (d, *J* = 3.5 Hz), 124.2, 119.4 (d, *J* = 277 Hz), 116.9, 115.6, 114.6 (d, *J* = 22.3 Hz), 101.9. HRMS calculated C_16_H_8_O_3_F_4_Na (M + Na^+^) 347.0307, found 347.0310.

#### 6.2.5. Methyl 2-((3-(4-fluorophenyl)-4-oxo-2-(trifluoromethyl)-4H-chromen-7-yl)oxy)acetate (**6**)

A solution of 4′-fluoro-7-hydroxy-2-trifluoromethylisoflavone (**5)** (0.35 g, 1.08 mmol) and potassium *tert*-butoxide (0.145 g, 1.29 mmol) in dry DMF (15 mL) was stirred at room temperature for 15 min under a nitrogen atmosphere. Thereafter, methyl bromoacetate (0.113 mL, 1.19 mmol) was added, and the mixture was stirred at room temperature for 6 h. The mixture was poured into ice water, neutralized with diluted HCl, and the yellow precipitate was filtered. The solid was subjected to flash chromatography to obtain the title compound **6** as a white solid (0.336 g, 0.85 mmol, 78.5%); Mp = 157–159 °C. FTIR (ν_max_/cm^−1^) 1750, 1661, 1617, 1508, 1448, 1203, 1200, 1136. ^1^H NMR (500 MHz, CDCl_3_) *δ_H_* 8.16 (d, *J* = 8.9 Hz, 1H, H-5), 7.23 (d, *J* = 8.7, 5.4 Hz, 2H, H-2′,6′), 7.14 (t, *J* = 8.7 Hz, 2H, H-3′,5′), 7.10 (dd, *J* = 8.9, 2.4 Hz, 1H, H-6), 6.91 (d, *J* = 2.4 Hz, 1H, H-8), 4.78 (s, 2H, -CH_2_-), 3.85 (s, 3H, CH_3_). ^19^F NMR (471 MHz, CDCl_3_) *δ_F_* −63.53, −112.66. ^13^C NMR (126 MHz, CDCl_3_) *δ_C_* 175.9, 168.0, 163.1 (d, *J* = 249 Hz), 163.0, 156.6, 148.3 (q, *J* = 36 Hz), 131.7 (d, *J* = 7.8 Hz), 128.2, 124.7 (q, *J* = 3.4 Hz), 124.7, 119.2 (q, *J* = 277 Hz), 117.8, 115.3, 115.4 (q, *J* = 22.1 Hz), 101.2, 65.3, 49.1. HRMS calculated C_19_H_12_O_5_F_4_Na (M + Na^+^) 419.0519, found 419.0508.

#### 6.2.6. 2-((3-(4-Fluorophenyl)-4-oxo-2-(trifluoromethyl)-4H-chromen-7-yl)oxy)acetic Acid **7**

Both **6** (150 mg, 0.378 mmol) and *p*-toluensulphonic acid (10 mg, 0.058 mmol) were added to formic acid 5 (mL) using the procedure described above for **4**, which afforded **7** as a white solid (118 mg, 0.38 mmol, 81.4%); Mp = 208–209 °C. FTIR (ν_max_/cm^−1^) 3466 br (COOH), 1708, 1648, 1611, 1435, 1253, 1208, 1166**.** ^1^H NMR (400 MHz, DMSO-*d_6_*) *δ_H_* 8.01 (d, *J* = 8.9 Hz, 1H, H-5), 7.40–7.25 (m, 5H, H2′,6′,3′,5′, 8), 7.20 (dd, *J* = 8.9, 2.4 Hz, 1H, H-6), 4.96 (s, 2H, -CH_2_-). ^19^F NMR (377 MHz, DMSO-*d_6_*) *δ_F_* −62.77 (s, 3F), −113.38 (s, 1F). ^13^C NMR (101 MHz, DMSO-*d_6_*) *δ_C_* 175.2, 169.4, 163.48, 163.3, 162.7 (d, *J* = 245 Hz), 156.3, 147.38, 147.0 (q, *J* = 35.5Hz), 132.08 (d, *J* = 8.3 Hz), 127.2, 125.7 (q, *J* = 3.5 Hz), 124.3, 119.3 (q, *J* = 275 Hz), 116.8, 116.3, 115.0 (q, *J* = 22.5 Hz), 101.6, 65.1. HRMS calculated C_18_H_10_O_5_F_4_Na (M + Na^+^) 405.0362 and found 405.0357.

### 6.3. Crystal Structure Determination

The crystallographic measurements for **2**, **5**, and **4** were accomplished as previously reported [38], where the CCDC in Table 6 represents crystal data and details of refinement. The crystallographic data were deposited with the Cambridge Crystallographic Data Centre as supplementary publication no. CCDC 2382565, 2382566, and 2382567, respectively. These data can be obtained free of charge from The Cambridge Crystallographic Data Centre via (www.ccdc.cam.ac.uk/structures).

### 6.4. Hirshfeld Surface Analysis

The topology analyses were performed using the Crystal Explorer 17.5 program [38].

The generation of Hirshfeld surfaces and 2D fingerprint plots around the primary molecule with all hydrates external was accomplished using Crystal Explorer 17.5. 

### 6.5. Anticancer Activity and Statistical Analysis

The methods of anticancer assessments and statistical analysis are reported in the Supplementary Information.

## 7. Conclusions

The fluorinated isoflavones 7-*O*-carboxymethyl-4′-fluoroisoflavone **4** and 7-*O*-carboxymethyl-4′-fluoro-2-trifluormethylisoflavone **7** were prepared in high yields starting from the corresponding deoxybenzoin **1**. Single-crystal X-ray diffraction was used to prove the molecular and supramolecular structures of the studied compounds. Compounds **2** and **4** were found to contain the ethanol molecule as the crystal solvent, which has a significant impact on the intermolecular interactions observed in these crystalline materials. The O…H interactions are more important in **2** and **4** than in **5**. Also, no interhalogen (F…F) contacts were detected in **2** and **7,** while both showed π-π stacking interactions. The opposite was true for **5**. Our investigation indicates that the synthesized isoflavones demonstrate significant anticancer activity against the MCF-7 cell line, corroborating the current literature regarding their effectiveness in hormone-dependent malignancies. The diminished activity shown against B16F10 melanoma cells indicated a selective effect that may be advantageous for targeting particular cancer types. The specificity of isoflavones against estrogen receptors or their high-binding affinity with these receptors seen on MCF-7 cells can be used to explain their notable and targeted action against breast cancer cells, including MCF-7, which is positive for ER alpha [39]. However, a feature of melanoma is the fact that it is resistant to many chemotherapeutic treatments, particularly when it is in an advanced stage. This wider resistance profile may be mirrored by the observed decreased influence of isoflavones; more research is required to elucidate this. Developing a better understanding of resistance mechanisms, such as immune evasion or the overexpression of survival pathways like NF-κB, can help to improve treatment approaches. Also, certain receptors or signaling pathways necessary for isoflavone-induced cytotoxicity could be absent in them, as many reports have mentioned [40]. Also, the isoflavones used in our study showed similar effects on MCF-7 cells, as their influences appeared to be closely matched. Notably, these compounds exhibit almost similar IC_50_ values (Table 6), indicating the same potency in inhibiting cell growth. Additionally, the selectivity index for each isoflavone is closely matched, suggesting that they all have a comparable ability to target cancerous cells while minimizing their impact on normal cells. This consistency in their bioactivity highlights their potential as promising therapeutic agents for further investigation and development. Finally, conducting in vivo and pre-clinical investigations will be essential to validate our results and comprehend isoflavones’ mechanisms of action and potential therapeutic applications. This research may substantially advance the field of cancer treatment.

## Figures and Tables

**Table 1:** Crystal data for the studied compounds.

Compound	2	5	4
CCDC	2382565	2382566	2382567
Empirical formula	C_16_H_13_FO_4_	C_16_H_8_O_3_F_4_	C_18_H_15_FO_6_
Formula weight	288.26	324.22	346.3
Temperature/K	100	173	173
Crystal system	monoclinic	orthorhombic	triclinic
Space group	*P2_1_/c*	*Pccn*	*P-1*
a/Å	6.3586(2)	26.4115(18)	9.2015(5)
b/Å	33.5685(13)	13.5418(11)	9.2999(5)
c/Å	6.7957(4)	7.7907(4)	9.8368(6)
α/°	90	90	89.288(5)
β/°	107.328(5)	90	71.493(5)
γ/°	90	90	86.818(5)
Volume/Å^3^	1384.70(11)	2786.4(3)	796.99(8)
Z	4	8	2
ρ_calc_g/cm^3^	1.383	1.546	1.443
μ/mm^−1^	0.108	1.248	0.991
F(000)	600	1312	360
Crystal size/mm^3^	0.2 × 0.04 × 0.02	0.14 × 0.08 × 0.015	0.14 × 0.06 × 0.04
Radiation	Mo Kα (λ = 0.71073)	Cu Kα (λ = 1.54184)	Cu Kα (λ = 1.54184)
2Θ range for data collection/°	4.854 to 58.114	6.694 to 163.266	9.482 to 133.29
Index ranges	−8 ≤ h ≤ 8, −43 ≤ k ≤ 45, −9 ≤ l ≤ 9	−30 ≤ h ≤ 32, −16 ≤ k ≤ 16, −9 ≤ l ≤ 9	−10 ≤ h ≤ 9, −9 ≤ k ≤ 11, −11 ≤ l ≤ 11
Reflections collected	27,429	25,327	14,066
Independent reflections	3374 [R_int_ = 0.0848, R_sigma_ = 0.0681]	2911 [R_int_ = 0.0847, R_sigma_ = 0.0312]	2774 [R_int_ = 0.0258, R_sigma_ = 0.0183]
Data/restraints/parameters	3374/0/193	2911/1/212	2774/2/235
Goodness-of-fit on F^2^	1.218	1.07	1.039
Final R indexes [I ≥ 2σ (I)]	R_1_ = 0.1001, wR_2_ = 0.1961	R_1_ = 0.0596, wR_2_ = 0.1810	R_1_ = 0.0350, wR_2_ = 0.0981
Final R indexes [all data]	R_1_ = 0.1396, wR_2_ = 0.2092	R_1_ = 0.0785, wR_2_ = 0.2073	R_1_ = 0.0371, wR_2_ = 0.1002
Largest diff. peak/hole/e Å^−3^	0.42/−0.31	0.28/−0.33	0.27/−0.23

**Table 2:** Bond lengths (Å) and angles (°) for **2**, **4,** and **5**.

**Bond**	**Length/Å**	**Bond**	**Length/Å**	**Bond**	**Length/Å**
**2**		**4**		**5**	
F4-C14	1.365(4)	F1-C11	1.328(3)	C7-C8	1.409(3)
O1-C2	1.346(4)	F2-C11	1.324(3)	C8-C9	1.381(3)
O1-C10	1.375(4)	F3-C11	1.327(3)	O1-C2	1.360(3)
O4-C4	1.251(4)	F4-C15	1.357(5)	O1-C10	1.379(3)
O8-C8	1.343(4)	O8-C8	1.356(3)	O4-C4	1.234(3)
**Bond**	**Length/Å**	**Bonds**	**Angle/**°	**Bonds**	**Angle/**°
C2-O1-C10	118.9(2)	C2-O1-C10	118.93(18)	F3-C11-C2	112.3(2)
C8-O8-H8	109.5(11)	C8-O8-H8	106.0(16)	F1-C11-F3	106.50(19)
O1-C2-C3	125.7(3)	O1-C2-C11	108.1(2)	C3-C12-C17	121.2(2)
C2-C3-C11	119.7(3)	C3-C2-C11	126.4(2)	C13-C12-C17	119.4(3)
C2-O1-C10	118.9(2)	C2-O1-C10	118.93(18)	F3-C11-C2	112.3(2)

**Table 3:** Hydrogen bonds for **2**, **4,** and **5** [Å and °].

D-H…A	d(D-H)	d(H…A)	d(D…A)	<(DHA)	Symm. Code
**2**					
O8-H8…O20	0.75(4)	1.91(4)	2.664(4)	175.7(16)	
O20-H20…O4	0.81(4)	1.91(3)	2.690(4)	162(2)	−1 + x,1/2 − y, −1/2 + z
C2-H2…O4	0.95	2.57	3.121(4)	117	−1 + x, y, z
**4**					
O10-H10…O21	0.960(19)	1.667(19)	2.5945(18)	161.2(18)	
O21-H21…O4	0.969(18)	1.704(18)	2.6674(14)	171.8(17)	1 + x, −1 + y, −1 + z
C13-H13…O4	0.95	2.46	3.3157(16)	150	−x, 1 − y, 2 – z
C16-H16…O9	0.95	2.59	3.2019(18)	123	−1 + x, 1 + y, z
**5**					
O8-H8…O4	0.97(2)	1.70(2)	2.664(3)	176.1(18)	−x, −1/2 + y, −1/2 − z
C7-H7…O4	0.95	2.38	3.092(3)	131	−x, −1/2 + y, −1/2 − z

**Table 4:** The important π-π contacts and their distances in **2**, **4,** and **5**.

2			4			5		
**C10…C10**	3.432(5)	x, 1/2 − y, −1/2 + z	**C16…C2**	3.384(2)	−x, 1 − y, 1 − z	**C4…C9**	3.418(3)	x, 1/2 − y, −1/2 + z
**C6…C6**	3.430(5)	x, 1/2 − y, −1/2 + z	**C2…C15**	3.328(2)	−x, 1 − y, 1 – z	**C5…C10**	3.464(3)	x, 1/2 − y, −1/2 + z
**C8…C4**	3.498(5)	x, 1/2 − y, −1/2 + z	**C8…C8**	3.430(2)	1 − x, −y, 1 − z	**C6…C10**	3.421(3)	x, 1/2 − y, −1/2 + z
**C7…C4**	3.408(5)	x, 1/2 − y, −1/2 + z	**C10…C4**	3.490(2)	1 − x, 1 − y, 1 − z	**C15…C16**	3.464(5)	x, 1.5 − y, −1/2 + z
			**C8…C16**	3.280(2)	1 − x, 1 − y, 1 − z			

**Table 5:** Interaction distances for the short contacts in the compounds studied.

Contact	Distance	Contact	Distance	Contact	Distance
2		4		5	
O4…H2	2.511	O8…H15	2.575	O4…H7	2.299
O1…H6	2.56	O9…H16	2.517	O4…H8	1.683
O20…H8	1.684	O4…H13	2.345	F1…F2	2.837
O20…H7	2.541	O4…H21	1.691		
O4…H20	1.745	O4…H13	2.345		
F4…H15	2.481	F1…H7	2.489		
		C2…C15	3.280		
		C2…C16	3.384		
		C8…C16	3.280		

**Table 6:** Anticancer activity of isoflavones.

Compounds #	IC50 (µM)/MCF-7	IC50 (µM)/MEF-1	SI (µM)
**7**	11.23	71.6	6.4
**4**	13.66	75.2	5.5
**5**	15.43	81.3	5.3
Daidzein	11.87	77.1	6.5

## Data Availability

The data will be made available upon request.

## Supplementary Materials

The supporting information can be downloaded at https://www.mdpi.com/article/10.3390/molecules30040795/s1. Figure S1. ^1^HNMR of **2**, Figure S2. ^13^CNMR of **2**, Figure S3. ^1^HNMR of **3**, Figure S4. ^19^FNMR of **3**, Figure S5. ^13^CNMR of **3**, Figure S6. ^1^HNMR of **4**, Figure S7. ^19^FNMR of **4**, Figure S8. ^13^CNMR of **4**, Figure S9. ^1^HNMR of **5**, Figure S10. ^19^FNMR of **5**, Figure S11. ^13^CNMR of **5**, Figure S12. ^1^HNMR of **6**, Figure S13. ^19^FNMR of **6**, Figure S14. ^13^CNMR of **6**, Figure S15. ^1^HNMR of **7**, Figure S16. ^19^FNMR of **7**, Figure S17. ^13^CNMR of **7.** References [34,35,36,39] are cited in the supplementary materials.
