# Supplementary material for: Efficient Consecutive Synthesis of Fluorinated Isoflavone Analogs, X-Ray Structures, Hirshfeld Analysis, and Anticancer Activity Assessment
Source: Molecules. 2025 Feb 9;30(4):795. doi: 10.3390/molecules30040795 (PMC11857983; doi:10.3390/molecules30040795)
Supplement: Supplementary file 1 [file molecules-30-00795-s001.zip › molecules-3442287-supplementary.pdf]

Supplementary Data

Efficient Consecutive Synthesis of Fluorinated Isoflavones Analogs, X-ray Structure, Hirshfeld Analysis and Anticancer Activity Assessment

Mohammed Salah Ayoup[1] *, Malak Daqa[2], Yousef Salama[3], Rand Hazzam[2], Mohammed B. Hawsawi [4], Saied M. Soliman,[5] and Nawaf Al-Maharik[2*]

1 Department of Chemistry, College of Science, King Faisal University, Al-Ahsa 31982, Saudi Arabia
2 Department of Chemistry, Science College, An-Najah National University, Nablus P.O. Box 7, Palestine
3 An-Najah Center for Cancer and Stem Cell Research, Faculty of Medicine and Health Sciences, An-Najah National University, P.O. Box 7, Nablus 00970, Palestine.
4 Department of Chemistry, Faculty of Science, Umm Al-Qura University, Al Taif Road, Makkah 24382, Saudi Arabia
5 Department of Chemistry, Faculty of Science, Alexandria University, Alexandria, Egypt.

* Correspondence: **mayoup@kfu.edu.sa & ***n.maharik@najah.edu (N.A.)*

Contents

1	Copy of ^{1}HNMR, ^{13}CNMR, ^{19}FNMR	**S2-S18**
2	Materials and Equipment	**S19**
3	Anticancer evaluation	**S19-S19**
4	Statistical Analysis	**S20**
5	References	**S20**

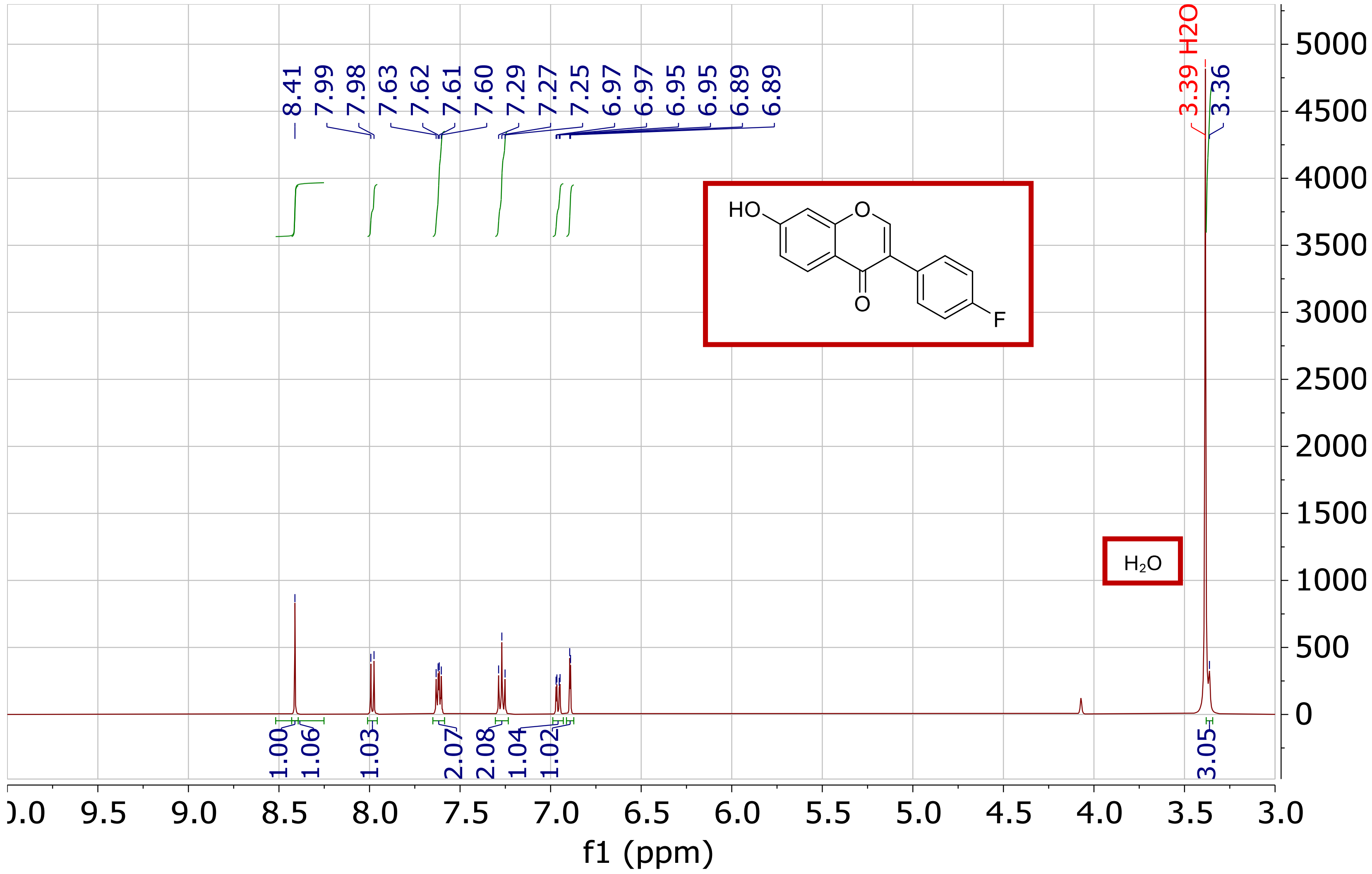

Figure S1. [1]**HNMR (500 MHz, DMSO-*d₆*) of 2**

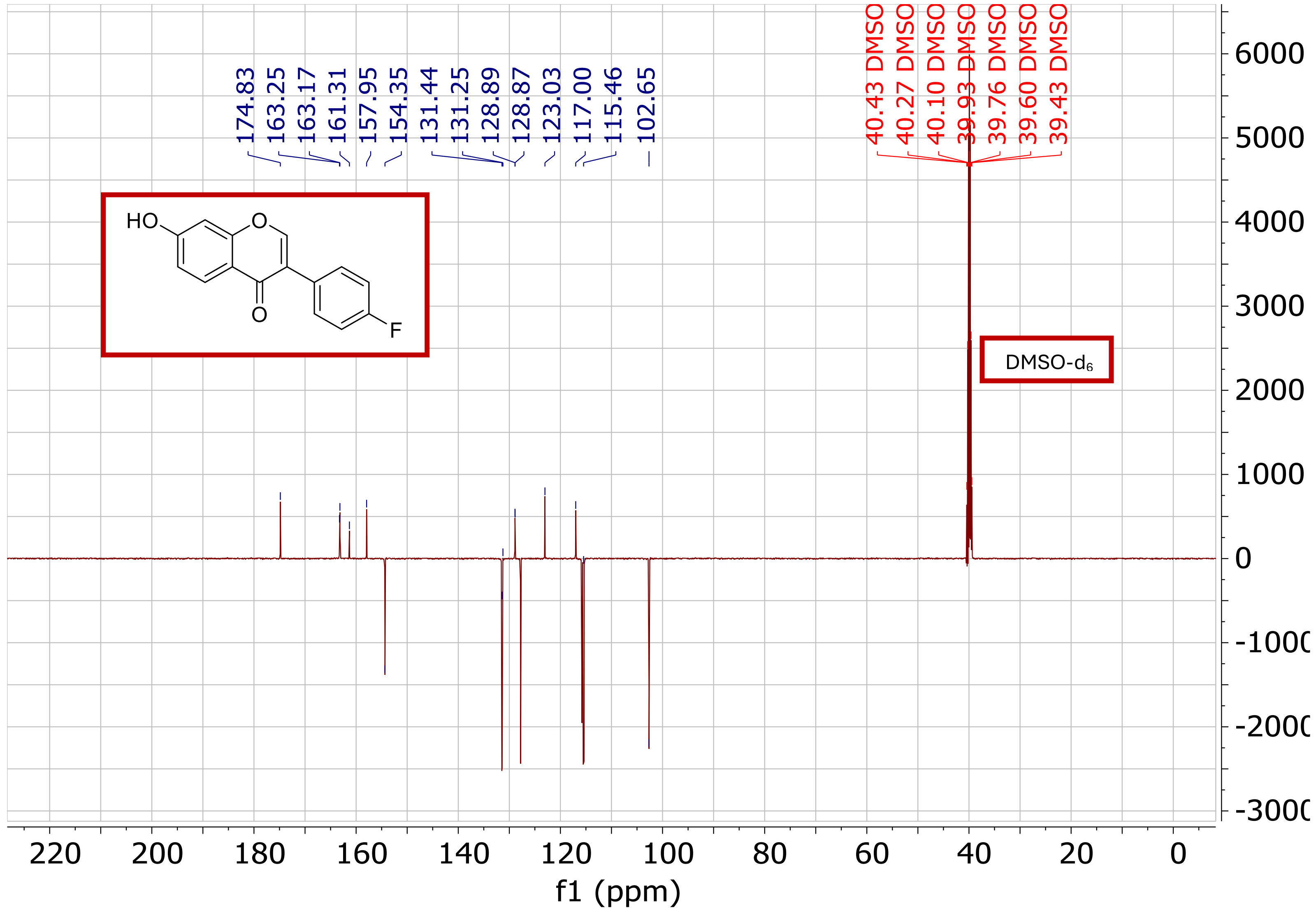

Figure S2. ^{13}**CNMR (126 MHz, DMSO-*d*$_6$) of 2**

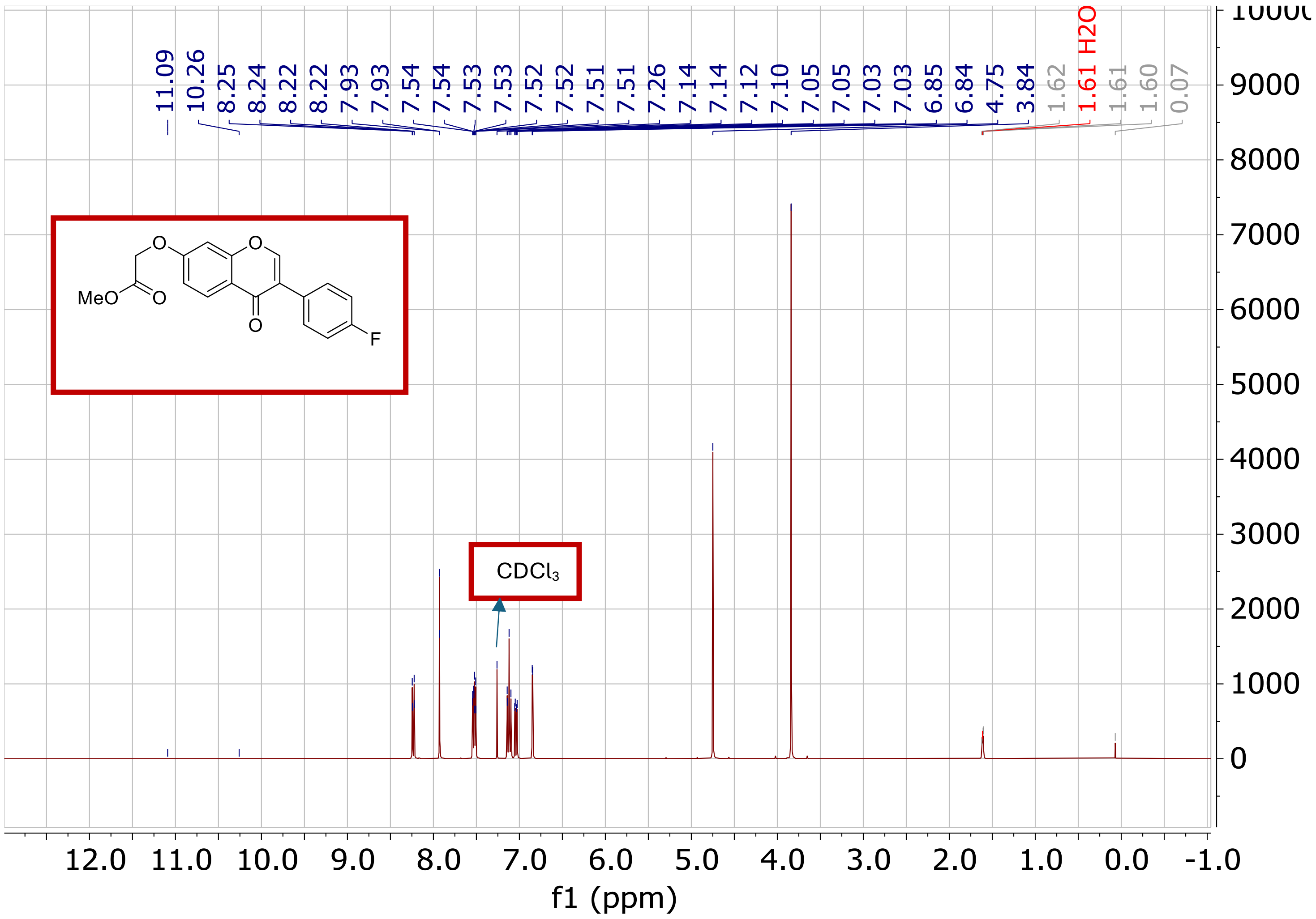

Figure S3. ¹HNMR (400 MHz, $CDCl_3$) of 3

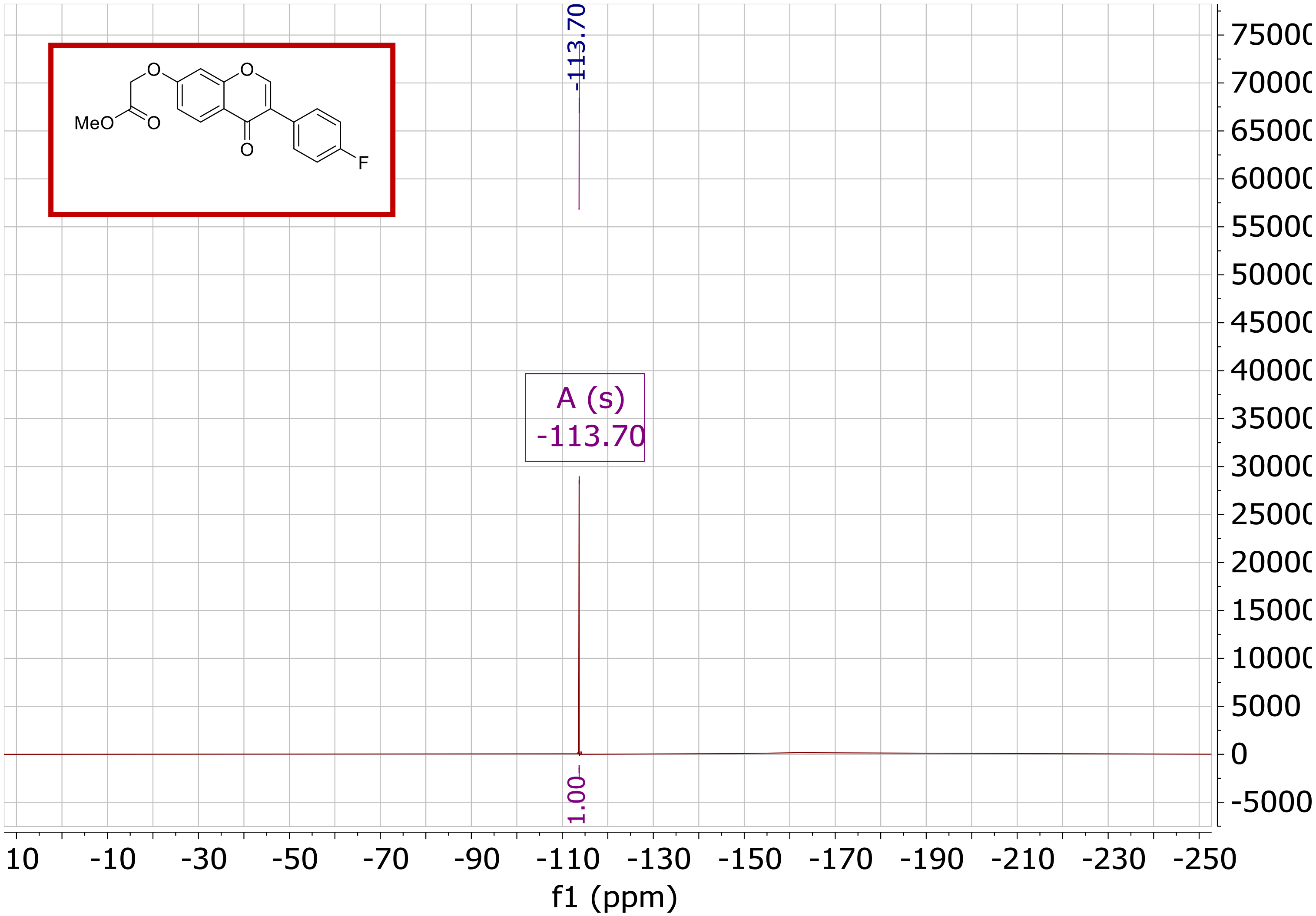

Figure S4. ^{19}FNMR (377 MHz, $CDCl_3$) of 3

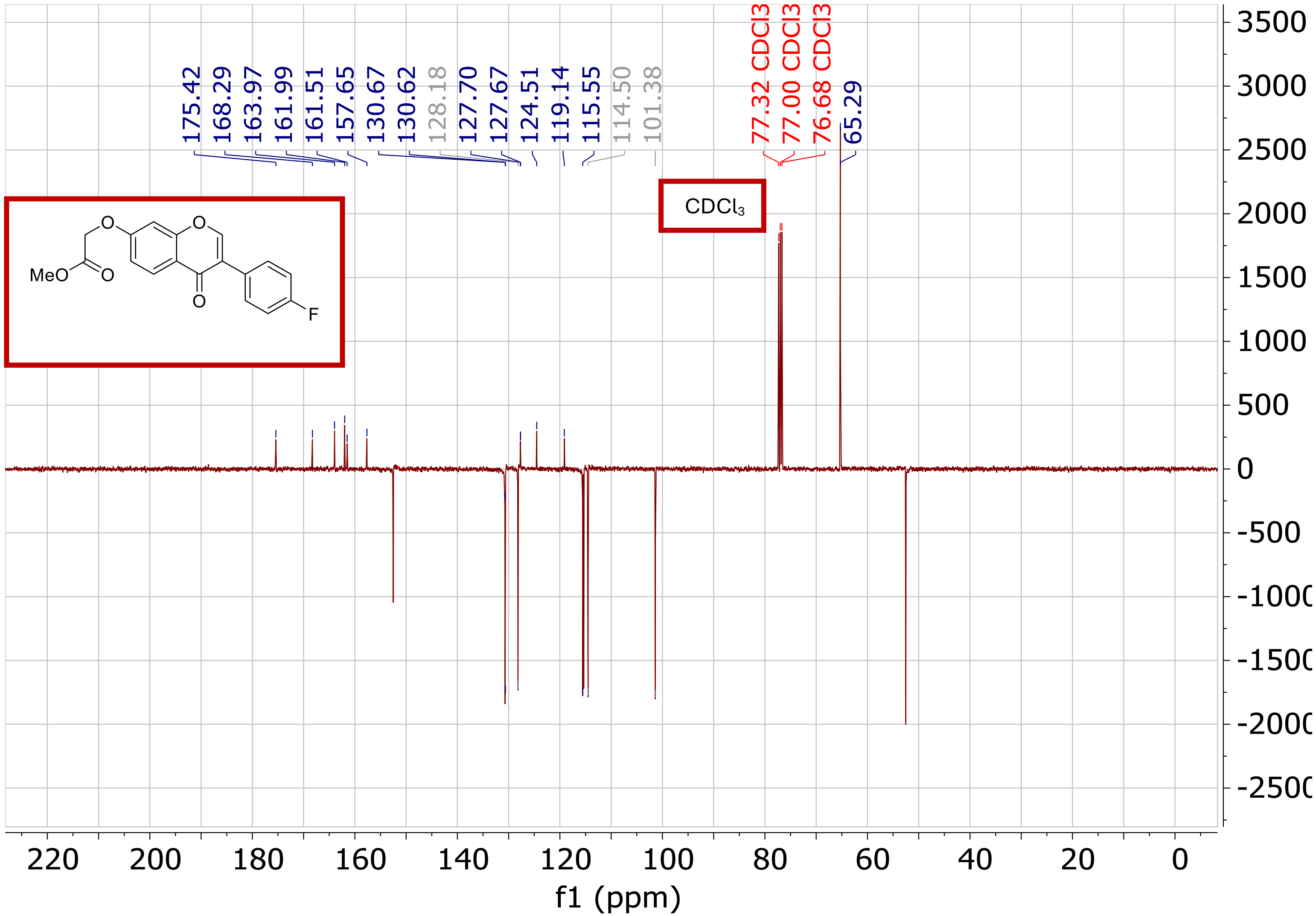

Figure S5. 13CNMR (101 MHz, $CDCl_3$) of 3

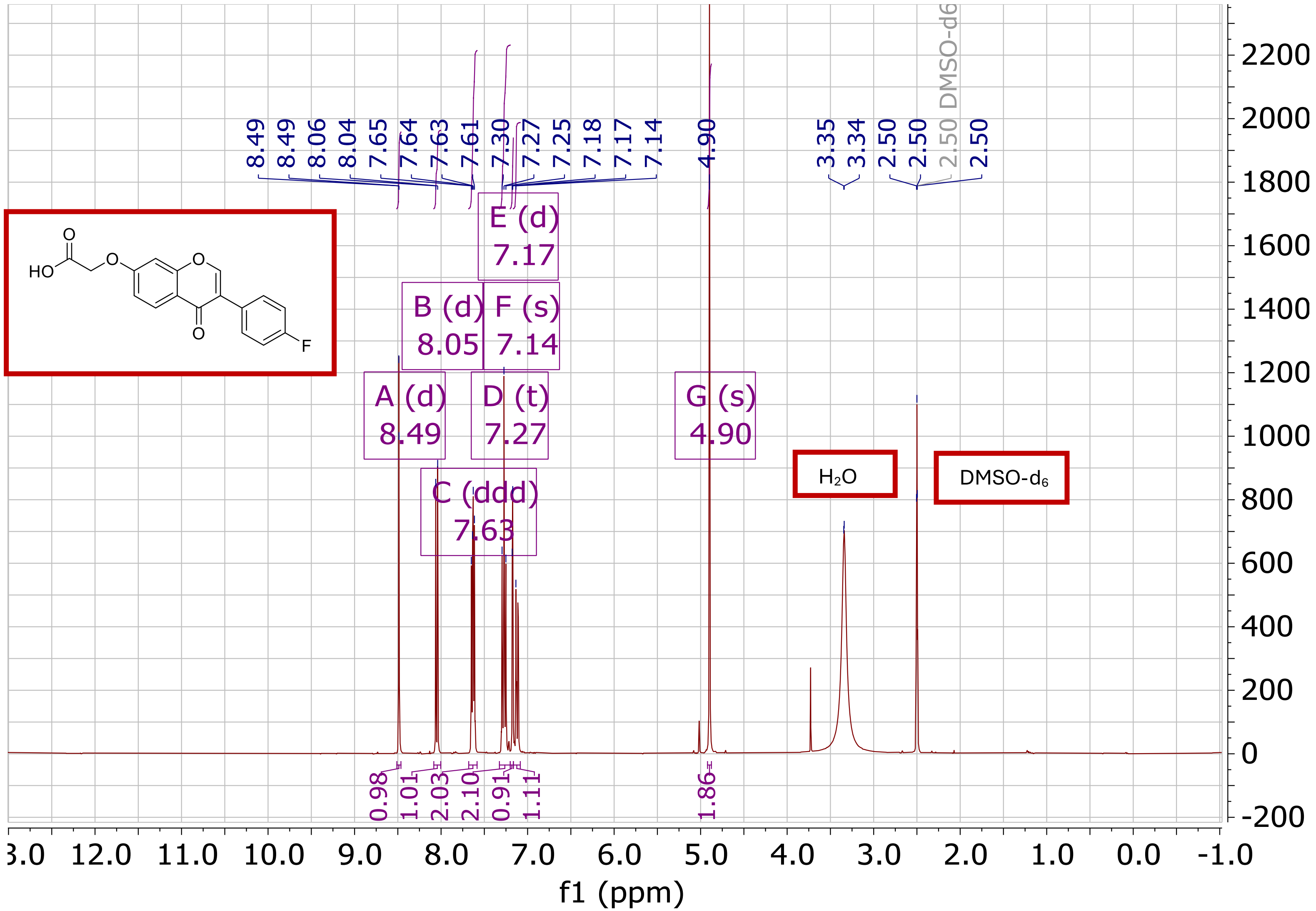

Figure S6. **^{1}HNMR (400 MHz, DMSO-*d*$_6$) of 4**

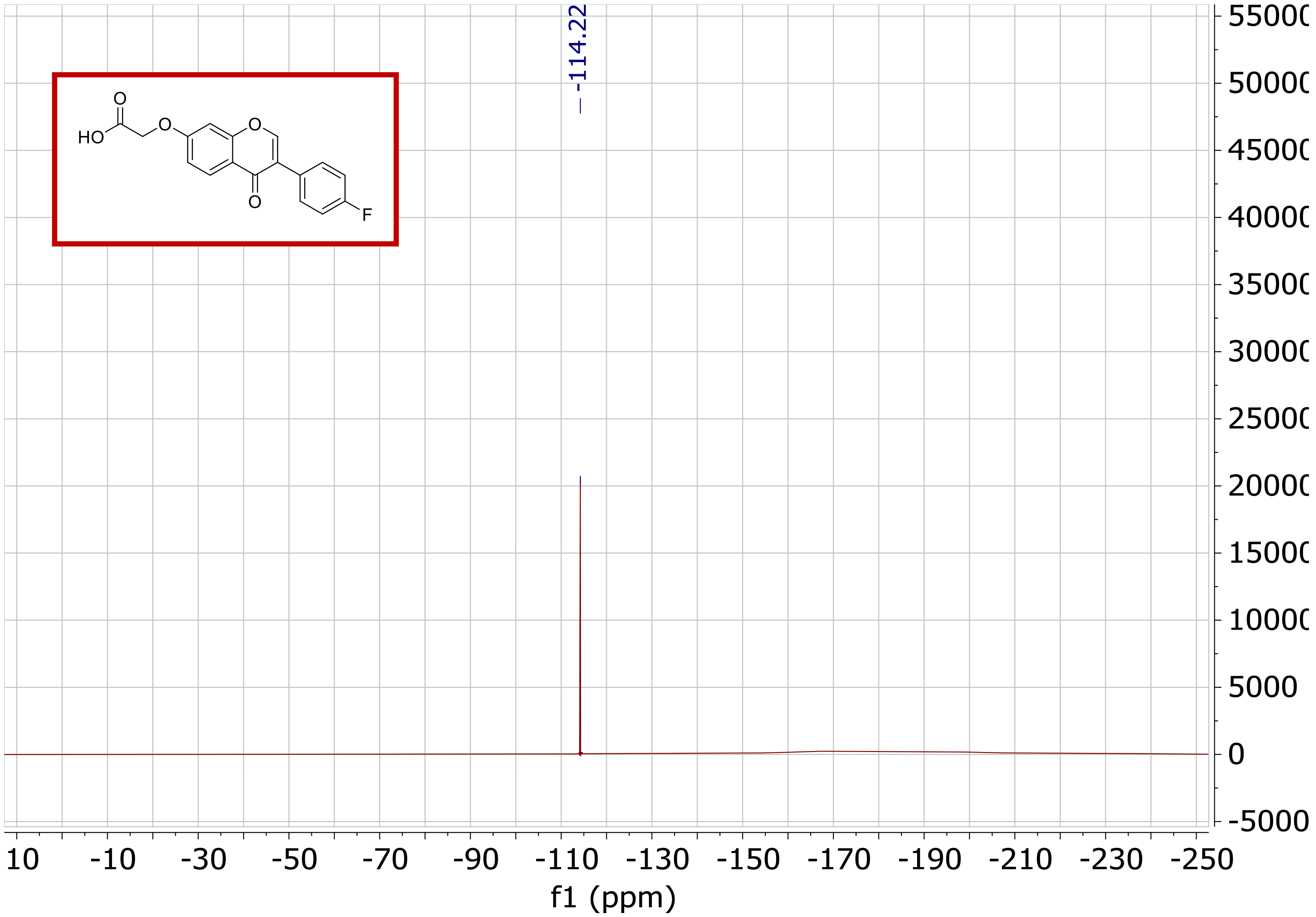

Figure S7. ^{19}FNMR (377 MHz, DMSO-d_6) of 4

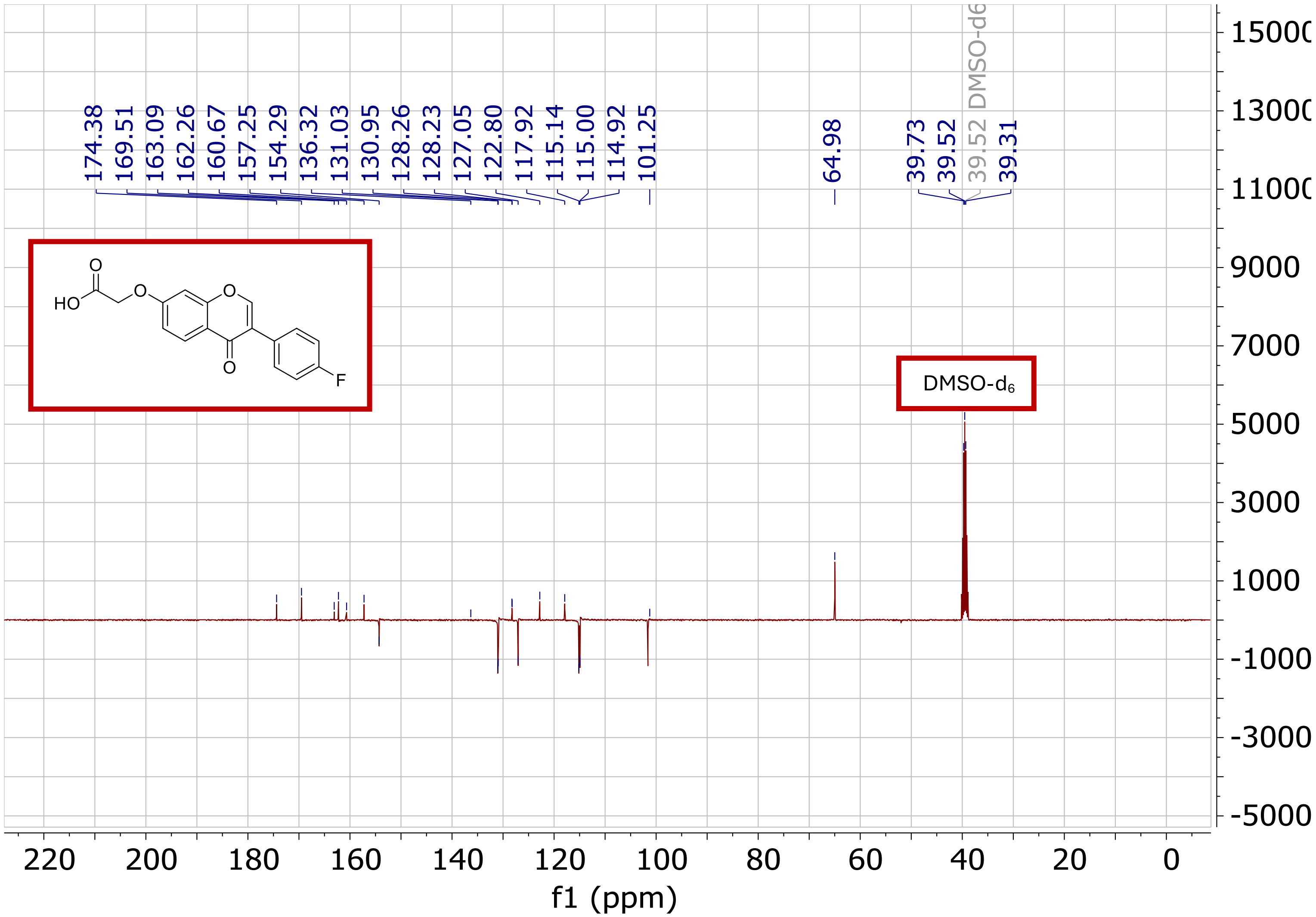

Figure S8. **^{13}CNMR (101 MHz, DMSO-d_6) of 4**

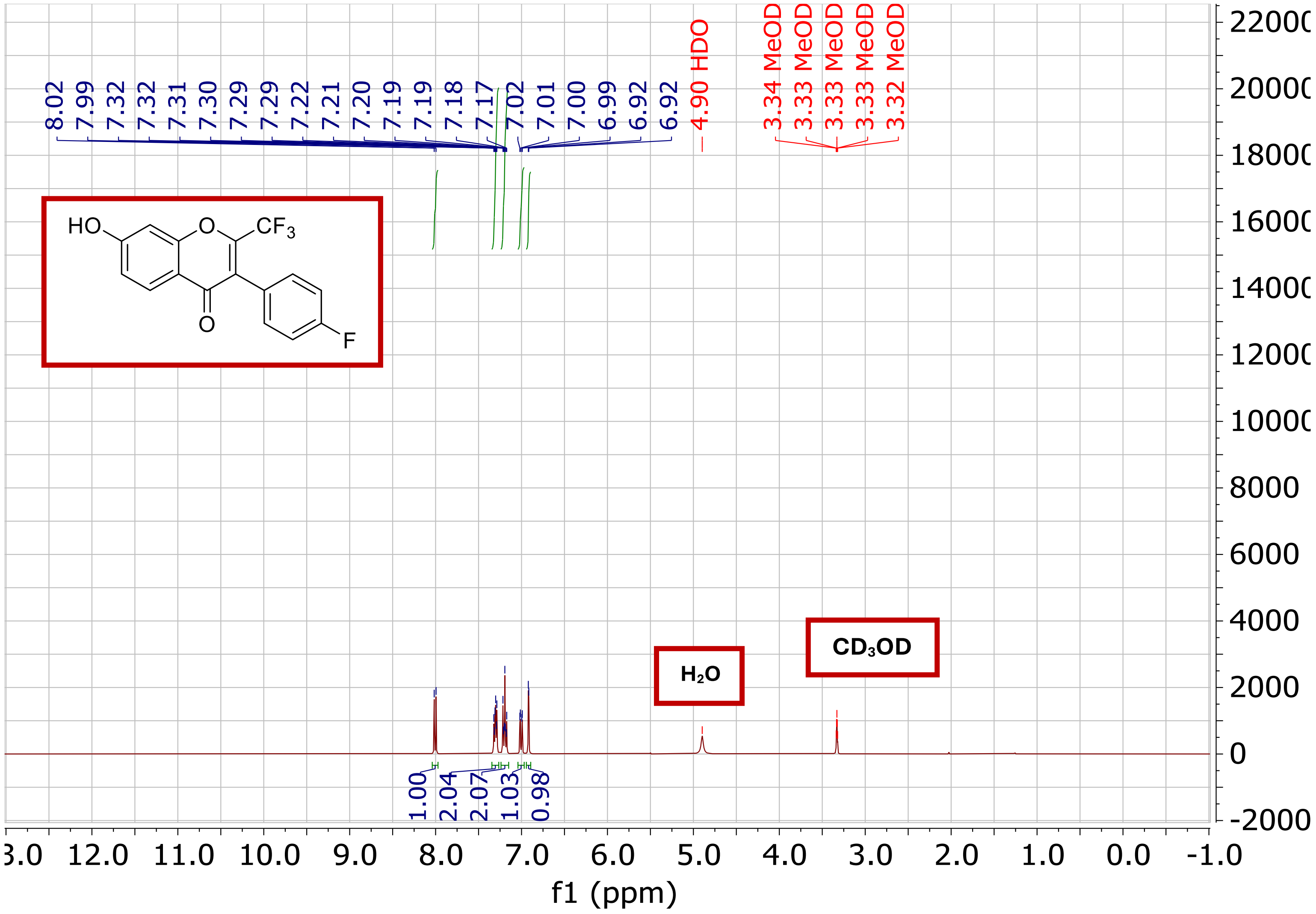

Figure S9. ^{1}**HNMR (400 MHz, CD_3OD) of 5**

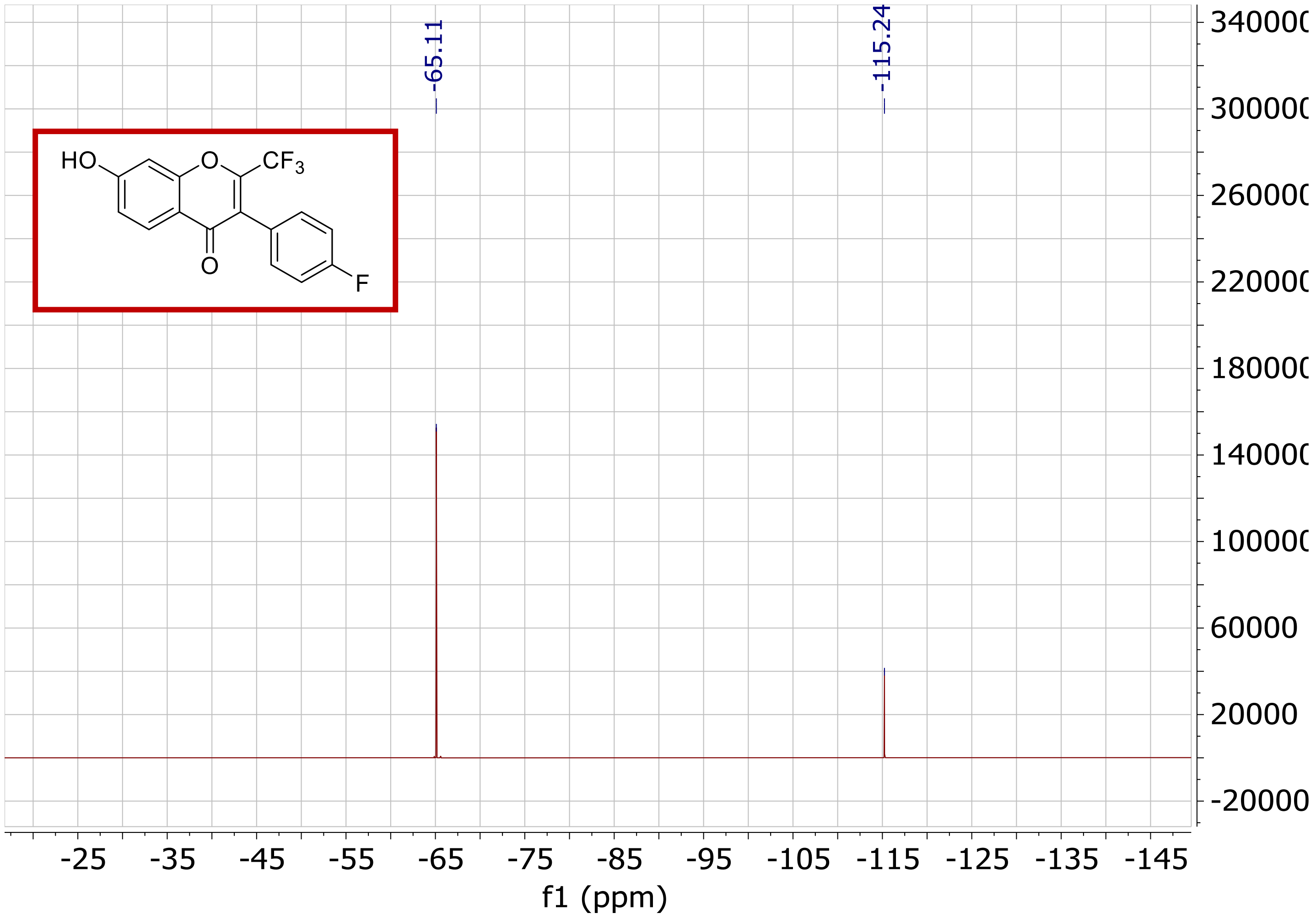

Figure S10. [19]**FNMR (377 MHz, CD_3OD) of 5**

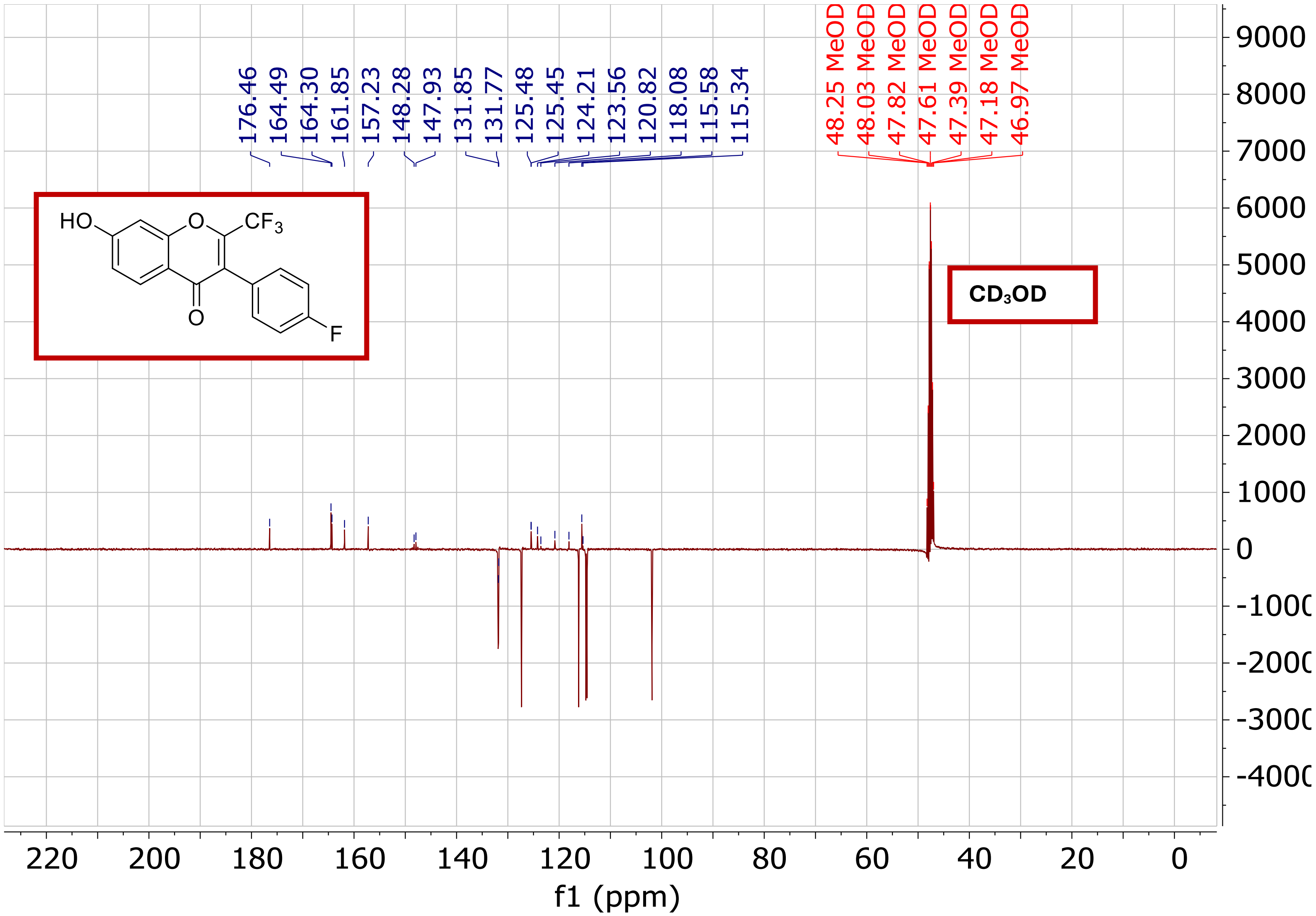

Figure S11. ^{13}CNMR (101 MHz, CD_3OD) of 5

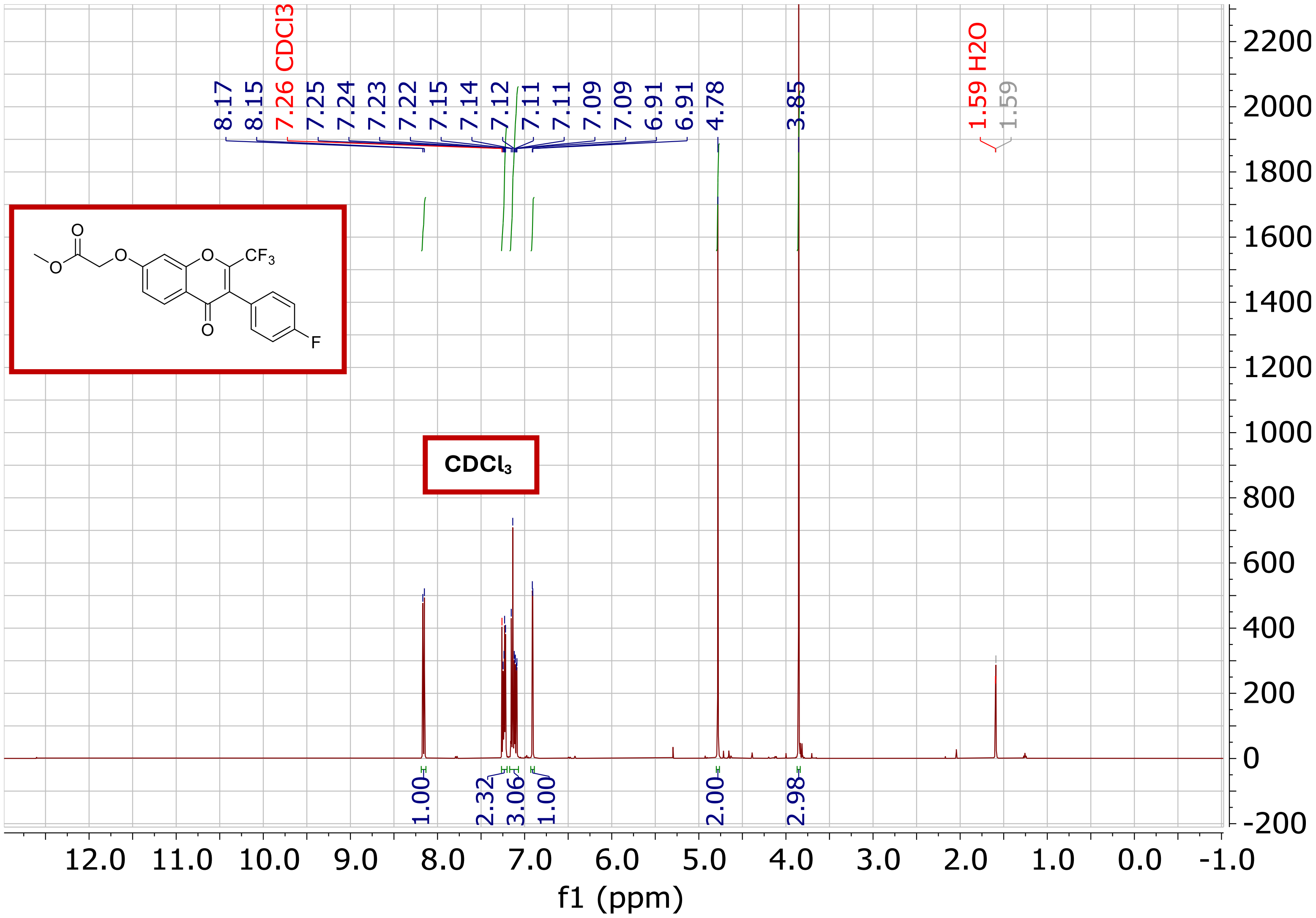

Figure S12. **^{1}HNMR (500 MHz, $CDCl_3$) of 6**

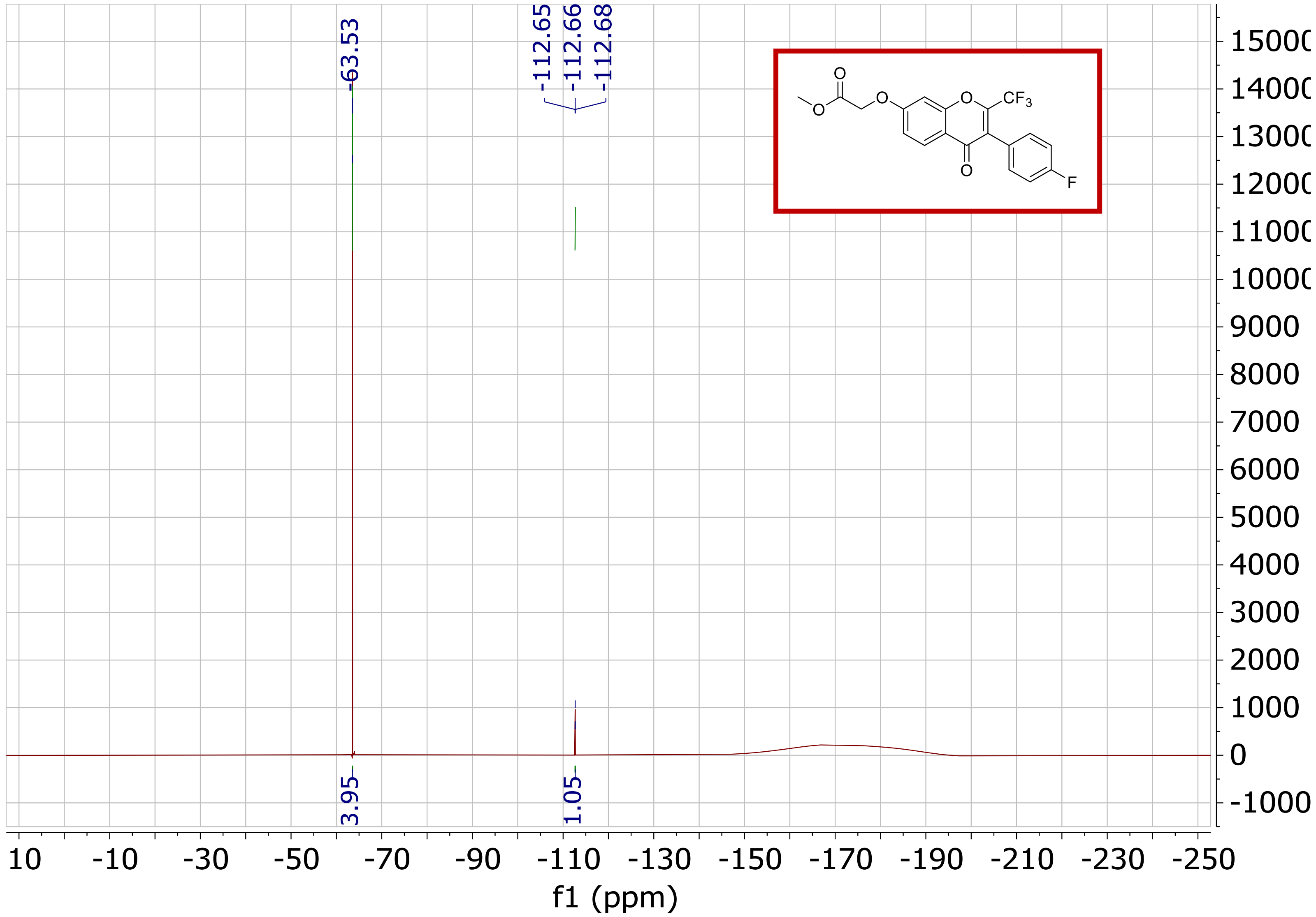

Figure S13. [19]**FNMR (471 MHz, $CDCl_3$) of 6**

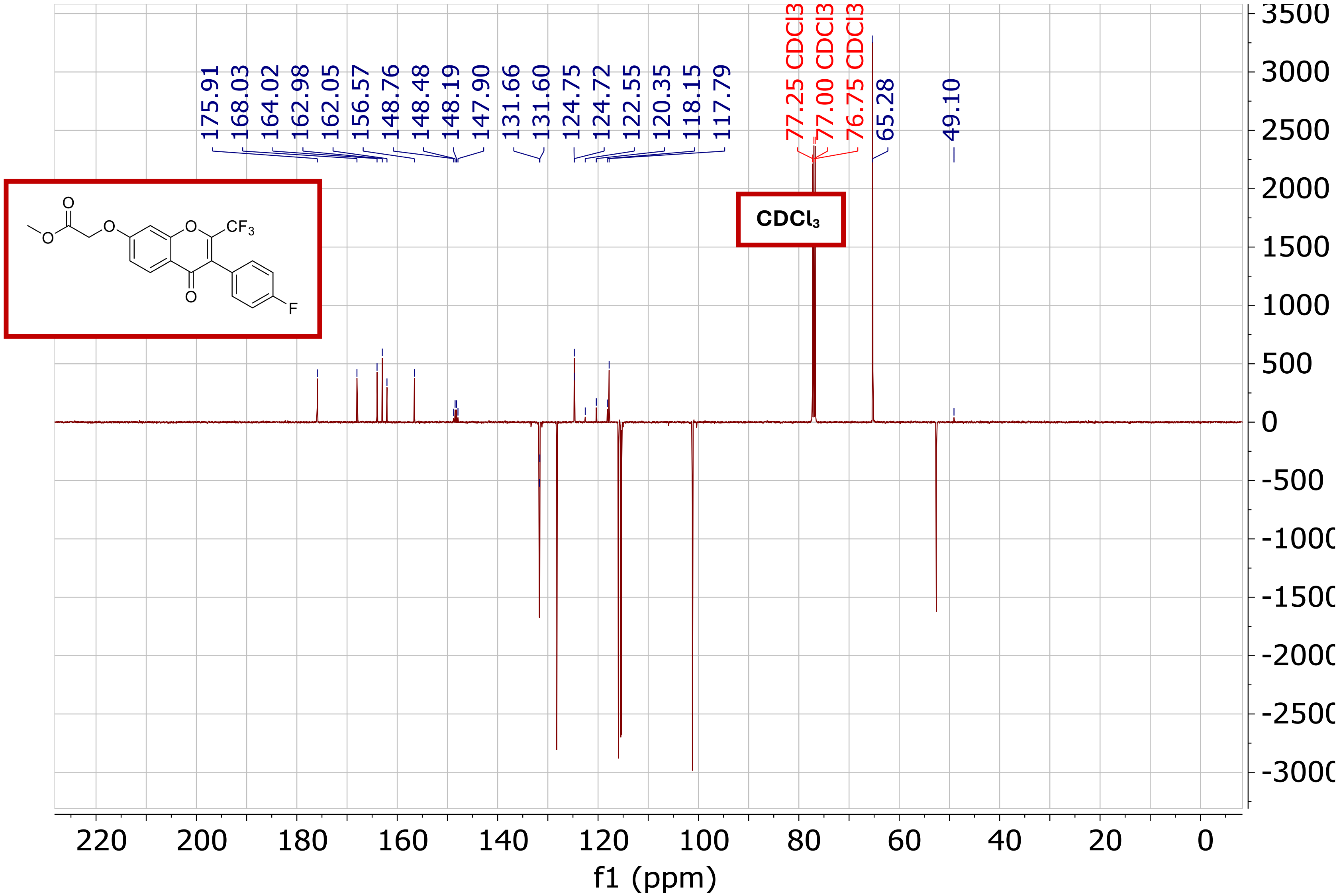

Figure S14. ^{13}**CNMR (126 MHz, CDCl$_3$) of 6**

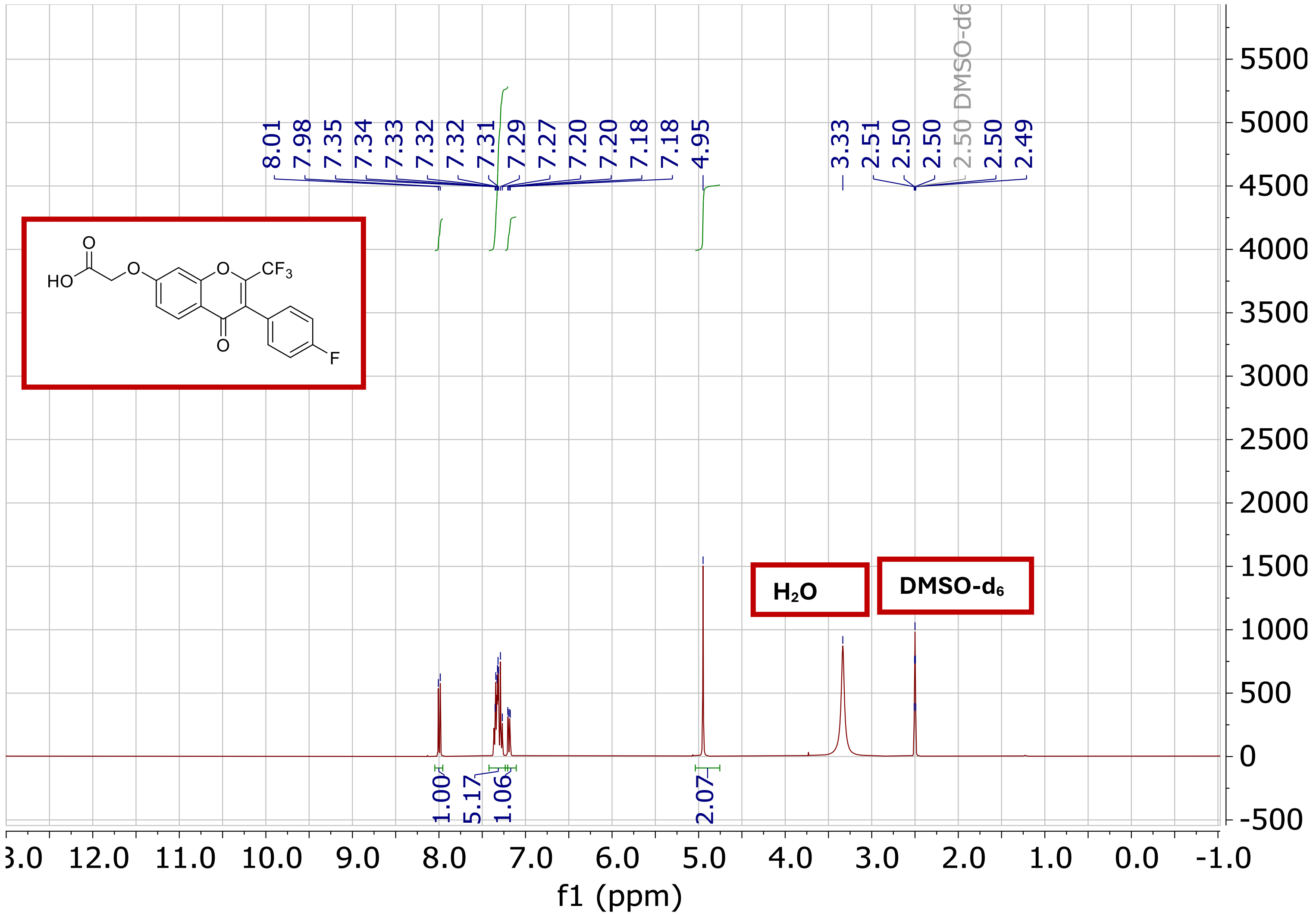

Figure S15. [1]**HNMR (400 MHz, DMSO-*d*$_6$) of 7**

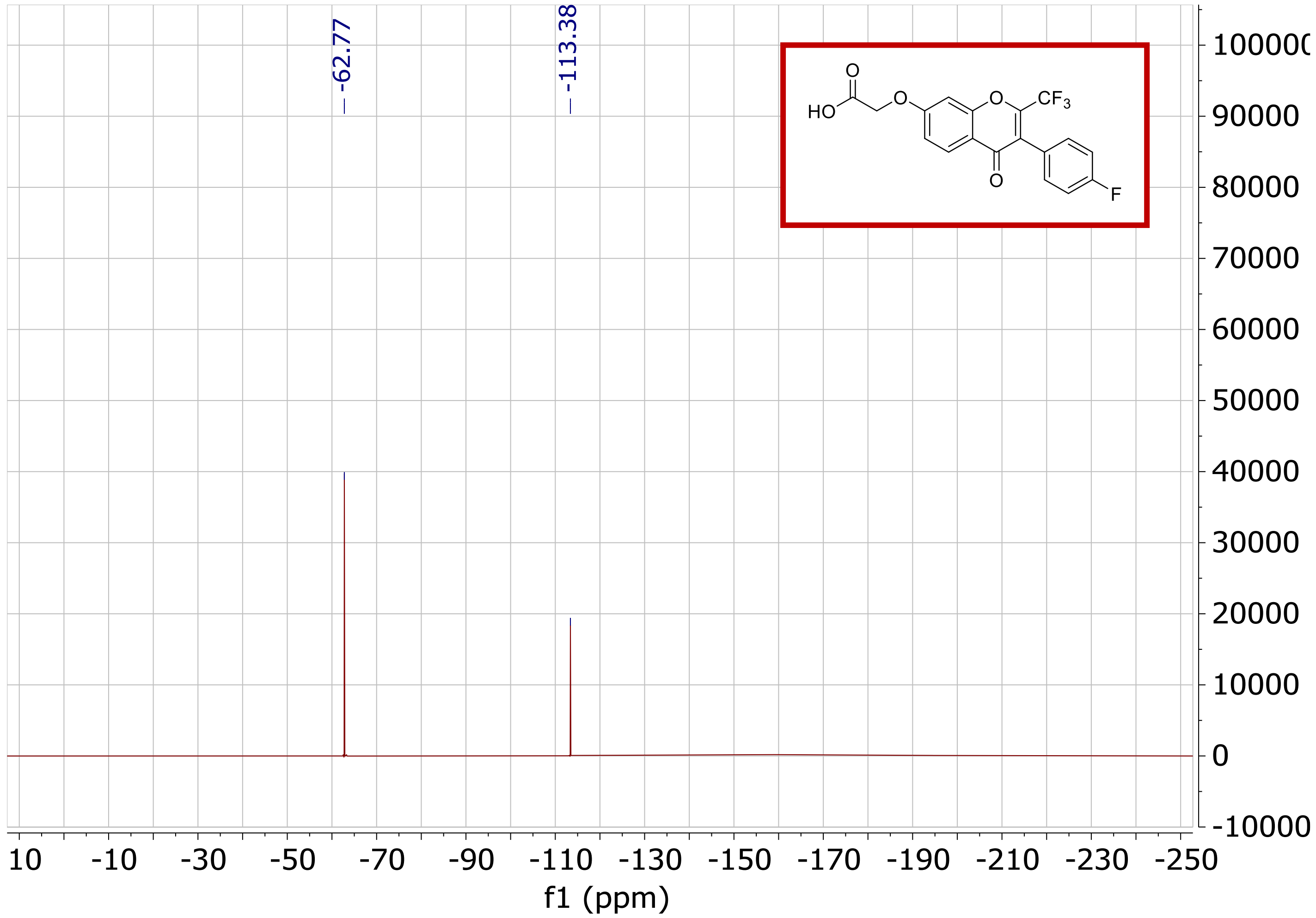

Figure S16. ^{19}**FNMR (377 MHz, DMSO-*d*$_6$) of 7**

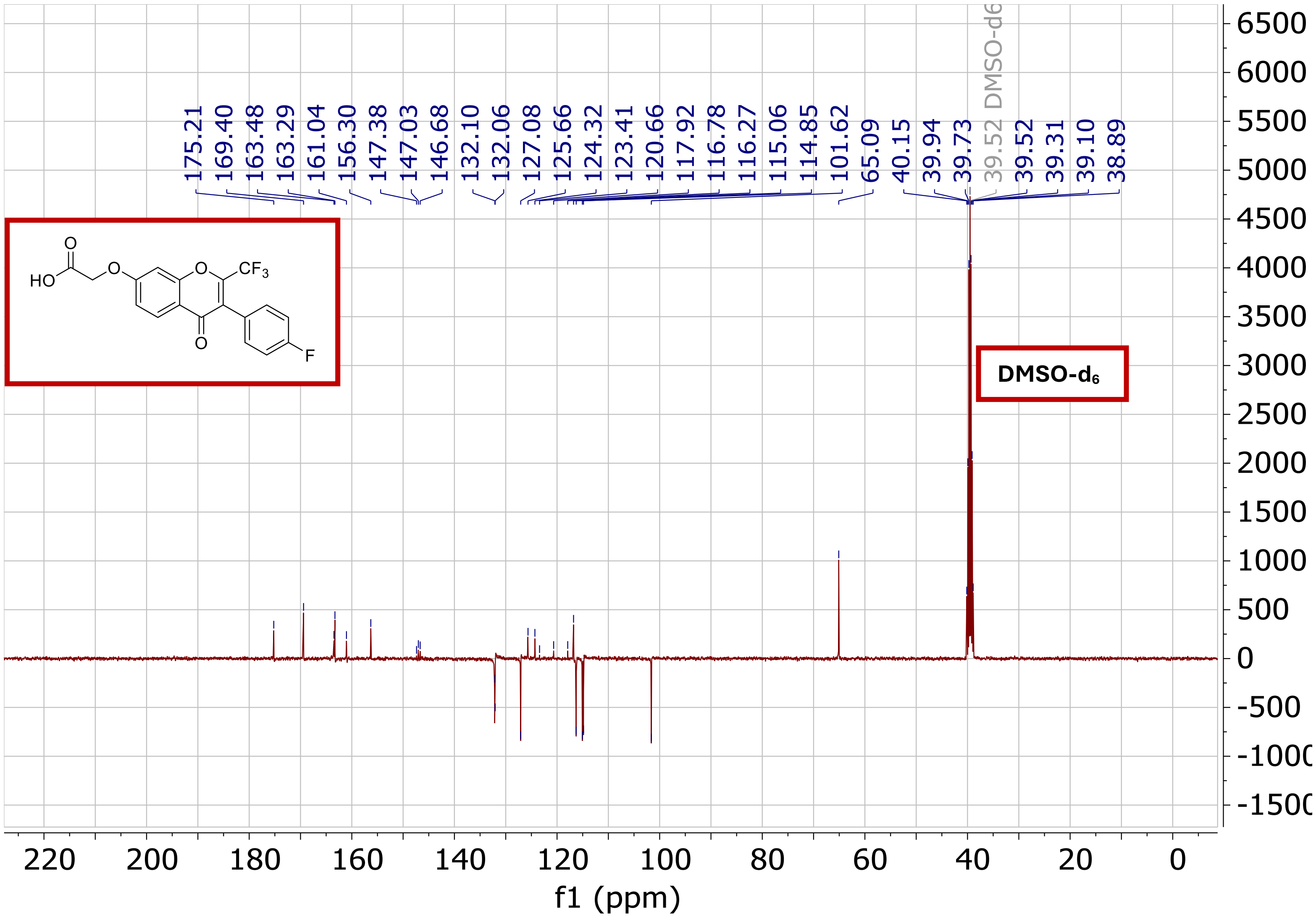

Figure S17. ^{13}**CNMR (101 MHz, DMSO-d_6) of 7**

.

3 Anticancer Evaluation

Cell lines

Human breast cancer cells MCF7 (ATCC number: HTB-22), mouse Embryonic Fibroblast-1(MEF-1; ATCC CRL-2214) and Murine melanoma B16F10 [CRL-6475; American Type Culture Collection (ATCC), Manassas, VA, USA] cell lines were maintained in Dulbecco's Modified Eagle's Medium (DMEM, high-glucose containing L-glutamine, phenol red (Sigma-Aldrich, Germany), 10% fetal bovine serum (FBS; G.E. Healthcare, Chicago, IL, USA), and 1% penicillin/streptomycin (P/S) (Nacalai Tesque, Kyoto, Japan) [34-36, 39].

Cell Cultures

MCF-7 (2 × 10^5 cells/well) and B16F10 (2 × 10^5 cells/well) cells were seeded in 6-well plates (TPP, Switzerland) and kept overnight before the addition of DMSO (control), Iso 3, Iso 4, or Iso 7 at concentrations ranging from 5-50 µM. Iso3, 4 and 7 were added in proliferation assays at 5µM, 25µM and 50µM. Viable cells were counted at 24 h using trypan blue (cat. 207-17081; Fuji Film Wako). Cell proliferation was also estimated using the MTT assay kit (Sigma, St. Louis, MO, USA).[[34-36, 39]

Cell proliferation assay

MCF-7 (2 x 10 5 cells/ml/well) and B16F10 (2 x 10 5 cells/ml/well) were cultured in triplicate in the presence of different concentrations of compounds for 24 hours. Trypan blue exclusion test was used to determine the cell viability. [34-36, 39]

Drugs treatment in vitro

A 10 mM stock solution was prepared in DMSO. A final concentration of (5, 25, and 50µM) was achieved by adding compounds into the cells. Cells were collected 24 hours after drug stimulation.

4 Statistical analysis

All experiments were performed at least three times. Data present the standard error of the mean (SEM). Student's t-test or ANOVA with Tukey HSD posthoc tests using the R program were used for statistical analysis. P-values < 0.05 were considered statistically significant.

5. References

34 Poschner, S., Maier-Salamon, A., Zehl, M., Wackerlig, J., Dobusch, D., Pachmann, B., Sterlini, K. L., & Jäger, W. (2017). The Impacts of Genistein and Daidzein on Estrogen Conjugations in Human Breast Cancer Cells: A Targeted Metabolomics Approach. *Frontiers in pharmacology*, *8*, 699. https://doi.org/10.3389/fphar.2017.00699

35 Sotoca, A. M., van den Berg, H., Vervoort, J., van der Saag, P., Ström, A., Gustafsson, J. A., Rietjens, I., & Murk, A. J. (2008). Influence of cellular ERalpha/ERbeta ratio on the ERalpha-agonist induced proliferation of human T47D breast cancer cells. *Toxicological sciences : an official journal of the Society of Toxicology*, *105*(2), 303–311. https://doi.org/10.1093/toxsci/kfn141

36 Hatono, M., Ikeda, H., Suzuki, Y., Kajiwara, Y., Kawada, K., Tsukioki, T., Kochi, M., Suzawa, K., Iwamoto, T., Yamamoto, H., Shien, T., Yamane, M., Taira, N., Doihara, H., & Toyooka, S. (2021). Effect of isoflavones on breast cancer cell development and their impact on breast cancer treatments. *Breast cancer research and treatment*, *185*(2), 307–316. https://doi.org/10.1007/s10549-020-05957-z

39 Kumar, V., & Chauhan, S. S. (2021). Daidzein Induces Intrinsic Pathway of Apoptosis along with ER α/β Ratio Alteration and ROS Production. *Asian Pacific journal of cancer prevention: APJCP*, *22*(2), 603–610. https://doi.org/10.31557/APJCP.2021.22.2.603
